# Substitution of Usual Perioperative Care by eHealth to Enhance Postoperative Recovery in Patients Undergoing General Surgical or Gynecological Procedures: Study Protocol of a Randomized Controlled Trial

**DOI:** 10.2196/resprot.6580

**Published:** 2016-12-21

**Authors:** Eva van der Meij, Judith AF Huirne, Esther VA Bouwsma, Johanna M van Dongen, Caroline B Terwee, Peter M van de Ven, Chantal M den Bakker, Suzan van der Meij, W Marchien van Baal, Wouter KG Leclercq, Peggy MAJ Geomini, Esther CJ Consten, Steven E Schraffordt Koops, Paul JM van Kesteren, Hein BAC Stockmann, A Dorien ten Cate, Paul HP Davids, Petrus C Scholten, Baukje van den Heuvel, Frederieke G Schaafsma, Wilhelmus JHJ Meijerink, H Jaap Bonjer, Johannes R Anema

**Affiliations:** ^1^ EMGO+ Institute for Health and Care Research Department of Public and Occupational Health VU University Medical Center Amsterdam Netherlands; ^2^ Department of Obstetrics and Gynaecology VU University Medical Center Amsterdam Netherlands; ^3^ Department of Obstetrics and Gynaecology Onze Lieve Vrouwe Gasthuis, Lokatie Oost Amsterdam Netherlands; ^4^ EMGO+ Institute for Health and Care Research Department of Health Sciences Vrije Universiteit, Faculty of Earth and Life Sciences Amsterdam Netherlands; ^5^ EMGO+ Institute for Health and Care Research Department of Epidemiology and Biostatistics VU University Medical Center Amsterdam Netherlands; ^6^ Department of Surgery VU University Medical Center Amsterdam Netherlands; ^7^ Department of Surgery Flevo Ziekenhuis Almere Netherlands; ^8^ Department of Obstetrics and Gynaecology Flevo Ziekenhuis Almere Netherlands; ^9^ Department of Surgery Maxima Medisch Centrum Veldhoven Netherlands; ^10^ Department of Obstetrics and Gynaecology Maxima Medisch Centrum Veldhoven Netherlands; ^11^ Department of Surgery Meander Medisch Centrum Amersfoort Netherlands; ^12^ Department of Obstetrics and Gynaecology Meander Medisch Centrum Amersfoort Netherlands; ^13^ Department of Surgery Spaarne Gasthuis Haarlem Netherlands; ^14^ Department of Obstetrics and Gynaecology Spaarne Gasthuis Haarlem Netherlands; ^15^ Department of Surgery Diakonessenhuis Utrecht Netherlands; ^16^ Department of Obstetrics and Gynaecology Diakonessenhuis Utrecht Netherlands; ^17^ Department of Surgery Jeroen Bosch Ziekenhuis Den Bosch Netherlands

**Keywords:** eHealth, mHealth, inguinal hernia surgery, cholecystectomy, adnexal surgery, perioperative care, convalescence, return to normal activities, cost-effectiveness, economic evaluation

## Abstract

**Background:**

Due to the strong reduction in the length of hospital stays in the last decade, the period of in-hospital postoperative care is limited. After discharge from the hospital, guidance and monitoring on recovery and resumption of (work) activities are usually not provided. As a consequence, return to normal activities and work after surgery is hampered, leading to a lower quality of life and higher costs due to productivity loss and increased health care consumption.

**Objective:**

With this study we aim to evaluate whether an eHealth care program can improve perioperative health care in patients undergoing commonly applied abdominal surgical procedures, leading to accelerated recovery and to a reduction in costs in comparison to usual care.

**Methods:**

This is a multicenter randomized, single-blinded, controlled trial. At least 308 patients between 18 and 75 years old who are on the waiting list for a laparoscopic cholecystectomy, inguinal hernia surgery, or laparoscopic adnexal surgery for a benign indication will be included. Patients will be randomized to an intervention or control group. The intervention group will have access to an innovative, perioperative eHealth care program. This intervention program consists of a website, mobile phone app, and activity tracker. It aims to improve patient self-management and empowerment by providing guidance to patients in the weeks before and after surgery. The control group will receive usual care and will have access to a nonintervention (standard) website consisting of the digital information brochure about the surgical procedure being performed. Patients are asked to complete questionnaires at 5 moments during the first 6 months after surgery. The primary outcome measure is time to return to normal activities based on a patient-specific set of 8 activities selected from the Patient-Reported Outcomes Measurement Information System (PROMIS) physical functioning item bank version 1.2. Secondary outcomes include social participation, self-rated health, duration of return to work, physical activity, length of recovery, pain intensity, and patient satisfaction. In addition, an economic evaluation alongside this randomized controlled trial will be performed from the societal and health care perspective. All statistical analyses will be conducted according to the intention-to-treat principle.

**Results:**

The enrollment of patients started in September 2015. The follow-up period will be completed in February 2017. Data cleaning and analyses have not begun as of the time this article was submitted.

**Conclusions:**

We hypothesize that patients receiving the intervention program will resume their normal activities sooner than patients in the control group and costs will be lower.

**ClinicalTrial:**

Netherlands Trial Registry NTC4699; http://www.trialregister.nl/trialreg/admin/rctview.asp?TC=4699 (Archived by WebCite at http://www.webcitation.org/6mcCBZmwy)

## Introduction

Between 1993 and 2013 the number of surgical procedures per year at community hospitals in the United States increased by 16.5% to more than 26 million per year [1]. This is partly due to the growing trend in day care surgery (ambulatory surgery). This is illustrated by the fact that in 2013 the number of surgeries performed in day care (17.4 million, 65.6%) exceeded the number of surgeries performed in overnight stay (9.1 million, 34.4%) [1]. Due to the strong reduction in the length of hospital stay, perioperative in-hospital care has been reduced accordingly. Once patients have been discharged, the degree of guidance and monitoring on recovery is limited, and sometimes conflicting advice is given by the different health care providers involved in the recovery process [2-5]. In addition, patients do not always know who to contact for support in case of postoperative complaints. This poor guidance and transition of perioperative care after hospital discharge contributes to patient uncertainties and postoperative fear that may hamper their recovery [6,7]. As a consequence, return to normal activities, including work, after surgery takes much longer than expected [8,9]. The delayed recovery has a negative impact on quality of life, clinical outcomes, and medical consumption and increases the risk of work disability, leading to an increased risk of mental health problems and poor general health [10,11]. In terms of the burden on society, this increases costs: costs resulting from the increased medical consumption (direct costs) and costs in relation to the high productivity loss due to prolonged sick leave (indirect costs) [10].

Therefore, improving the quality of perioperative care may contribute to accelerated recovery and health care efficiency, which in turn may reduce health care costs. eHealth seems to be an effective tool in this process for several reasons. First, electronic devices are widely available and are increasingly popular. This means that patients can be easily reached using this medium. Second, there is an increasing demand for self-management in society; eHealth has the potential to motivate people and turn them into more active and effective managers of their own health [12,13]. Finally, eHealth is a useful tool to provide the patients with tailored information, by providing only advice based on the patient’s profile. Therefore an eHealth care program has been developed to improve perioperative care after gynecological surgery [14]. The program was developed using an intervention mapping protocol, based on a systemic review of the literature, input of patients during focus groups, and consented guidelines on resumption of activities after surgery achieved after a Delphi procedure [14,15]. The effect of the eHealth care program on return to work was evaluated in a randomized controlled trial (RCT). Patients who had access to the care program returned to work 9 days earlier than patients from the control group [16]. Because of these promising results, the care program was further developed for a broader population, according to the wishes and preferences of a sample of patients who had undergone various types of abdominal surgery. While the gynecological care program aimed to deliver additional care by providing extra support and information through eHealth, this adapted care program also aims to partly substitute perioperative care with eHealth. In addition, the care program will focus on health-behavior change techniques using an activity tracker, which has been described before as an effective strategy [17,18]. With this multicenter, single-blinded, RCT, we will evaluate whether this adapted perioperative eHealth program will be effective and cost effective as compared to usual care on the resumption of normal activities in patients undergoing commonly applied minor abdominal surgical procedures with a short duration of hospital stay.

## Methods

### Study Setting

Patients will be included from the surgical and gynecological departments of 7 teaching hospitals in the Netherlands. The trial was conducted in accordance with the Standard Protocol Items: Recommendations for Interventional Trials and reported in accordance with the Consolidated Standards of Reporting Trials [19-21]. The study was approved by the local medical ethics committee under registration number 2014.301 and by the institutional review boards of all participating hospitals. The study is registered at the Netherlands Trial Registry (NTR4699).

### Eligibility Criteria

Eligible patients for this study are adults from 18 to 75 years old who are on the waiting list for one of the following commonly applied minor surgical procedures: laparoscopic cholecystectomy, open or laparoscopic inguinal hernia surgery, or laparoscopic adnexal surgery. Participants meeting any of the exclusion criteria will not be considered (Textbox 1).

**Exclusion criteria.**

Exclusion criteria:

- Surgery without a curative intention or with additional radio- or chemotherapy
- Deep infiltrating endometriosis
- Ectopic pregnancy
- Adnexal surgery because of pelvic inflammatory disease or tubal ovarian abscess
- Combination of several surgical procedures
- Severe comorbidity which might complicate the postoperative course
- Patient who are unable to understand the information from the study
- Insufficient understanding or ability to complete questionnaires in Dutch

### Interventions

#### Control Group

Patients allocated to the control group will receive usual care and access to the nonintervention part of the website (www.ikherstel.nl). On this part of the website, the patient information brochure about the surgical procedure from the hospital where the patient will have surgery is presented. This is the same brochure as the one patients often receive in various hospitals when they are scheduled for surgery. The only reason to give patients access to this website is to minimize the bias in estimation of the intervention effect. Furthermore, pre- and postoperative care will be given according to the local protocol of the hospital. In the Netherlands, patients do not receive structural and detailed instructions about the resumption of normal activities including work. Usually, after the patient is discharged from the hospital, an outpatient postoperative consultation is scheduled 4 to 8 weeks following surgery.

#### Intervention Group

Patients in the intervention group will receive access to the intervention part of the website, a mobile application, and an activity tracker. Table 1 provides an overview of the different components of the intervention.

**Table 1:** Components of the intervention.

Component	Target population	Content	Aim
Website	All patients of the intervention group	Information by text and animations^a^	Enhancing patient involvement and recovery expectations and reducing anxiety
		Making a personalized convalescence plan^a^	Creating recovery expectations and improving recovery
		Recovery monitor and recovery report^a^	Reducing uncertainties and fear related to the recovery process and improving monitoring of postoperative care
		eConsult	Increasing access to care, reducing patient uncertainties and fear related to the recovery process, and reducing costs and workload (by replacing the appointment in the outpatient clinic)
App	All patients of the intervention group with a smartphone	Information by text^a^	Enhancing patient involvement and recovery expectations and reducing anxiety
		Insight in the convalescence plan^a^	Creating recovery expectations and improving recovery
		Recovery monitor and recovery report^a^	Reducing uncertainties and fear related to the recovery process and improving monitoring and transition of postoperative care
		Creating a packing list	Helpful tool
		Section to make notes	Helpful tool
Activity tracker	All patients of the intervention group with a smartphone which can be linked to an activity tracker	Monitoring and giving feedback on recovery^a^	Reducing uncertainties and fear related to the recovery process, which may improve recovery

^a^Content is based on the intervention mapping study of Vonk Noordegraaf et al [14].

##### Website

The website aims to prepare patients in the best possible manner for their surgery and to offer guidance during their recovery process until full recovery and resumption of all daily activities are achieved. The following tools on the website will support this.

###### Providing Information About the Surgical Procedure and Recovery Process

**Figure 1:**
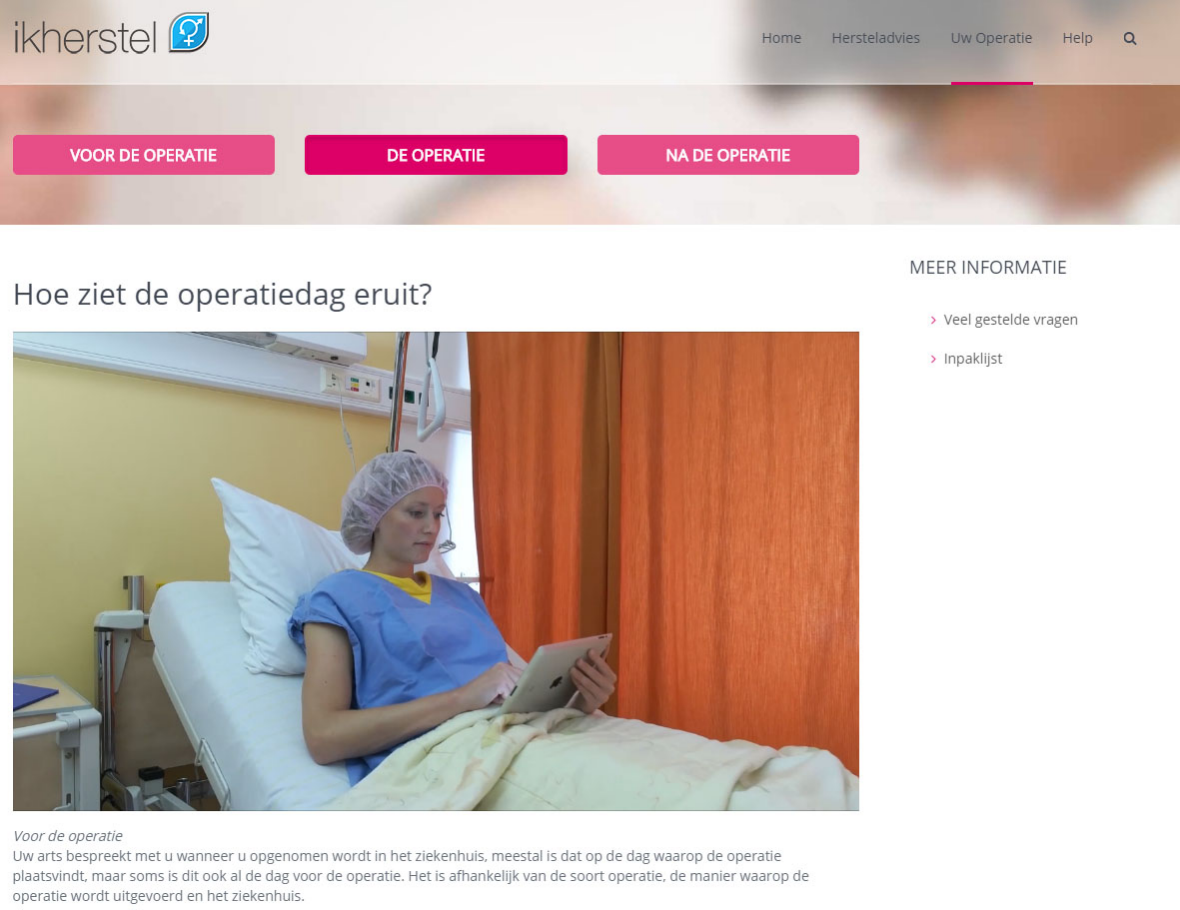
Text and animations on the website (see Multimedia Appendix 1 for more information).

On the website, information will be tailored to the patient, which offers the opportunity to enhance patient involvement (Figure 1) [13]. This is possible because some data are already prefilled when patients receive their website account (eg, surgical procedure, sex, hospital). The information will be offered by text as well as animations. The aim is to prepare the patient as well as possible for the day of surgery, which may contribute to the patient being more aware of what to expect and have a positive effect on anxiety and satisfaction [22]. In addition, it aims to improve recovery expectations, as expectations about the length of the recovery before surgery have proven to be an important predictor of the length of recovery [23]. After surgery, text and animations will be offered about the recovery period, which may support patients during this period and may help them with feelings of insecurity. Information about several postoperative complaints is available, including practical advice about when, how, and with whom patients should seek contact. We hypothesize that by providing this information, patients will be encouraged to resume their daily activities. In addition, this empowerment will help when deciding whether contact with a health care provider is indicated in case of complaints or complications during their recovery.

###### Making a Personalized Convalescence Plan

**Figure 2:**
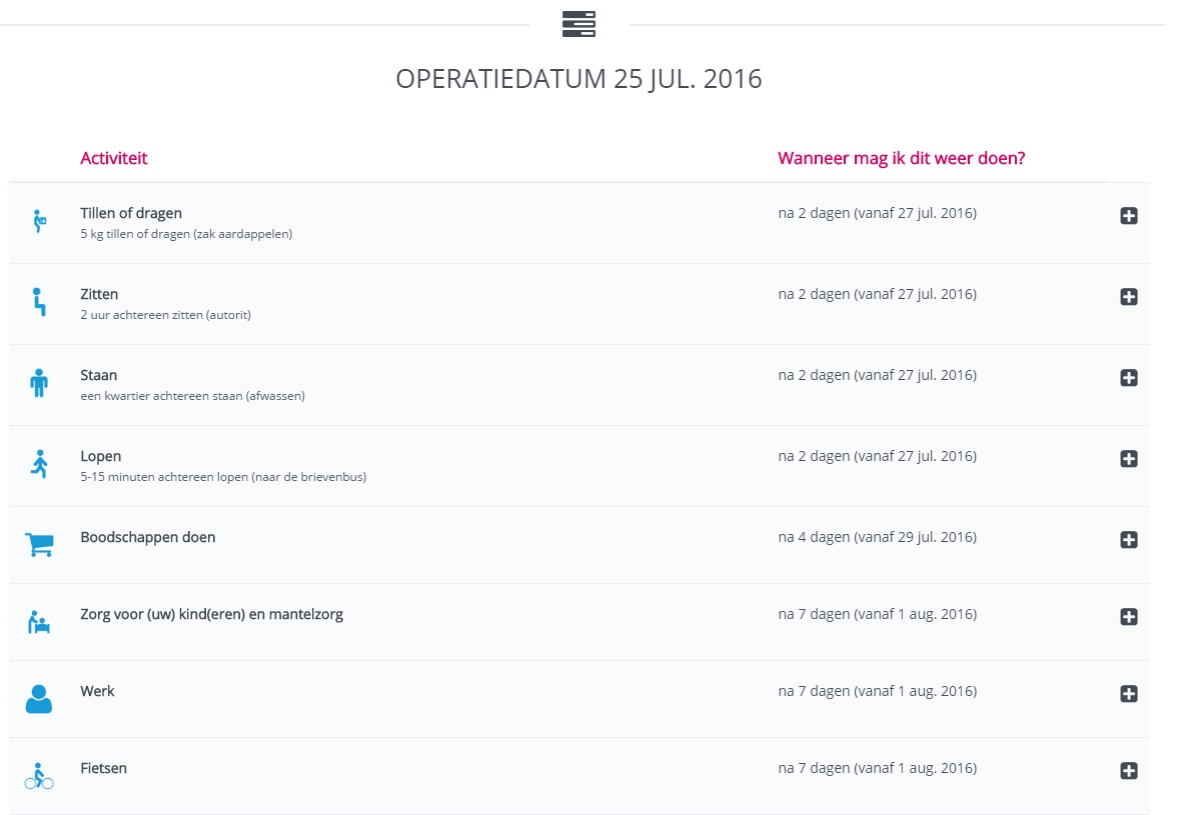
Personalized convalescence plan (see Multimedia Appendix 1 for more information).

**Figure 3:**
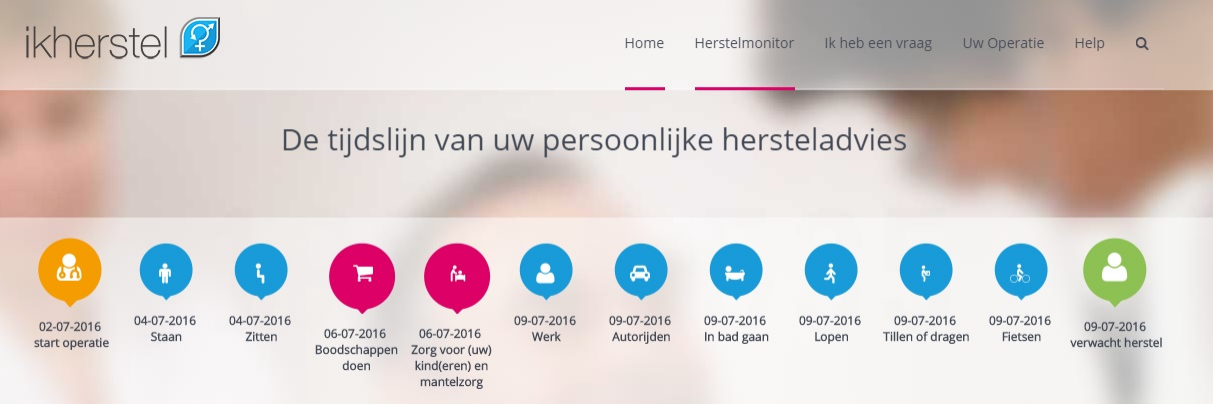
Personalized convalescence plan displayed on a timeline (see Multimedia Appendix 1 for more information).

The most important tool on the website is the option to generate a personalized and tailored convalescence plan, including advice about resumption of daily (work) activities (Figures 2 and 3). Using a modified Delphi method, specific convalescence recommendations were developed for several types of abdominal surgical procedures [24,25]. The recommendations given are tailored to the patient and are based on the input of patients’ normal preoperative activities and the surgical technique (using algorithms). It aims to improve recovery, return to normal (work) activities, and quality of life. The convalescence plan will be approved electronically on the first postoperative day by the operating surgeon, resulting in a definitive convalescence plan. If complications occur during surgery, the surgeon will not approve the convalescence plan and the patient will receive a message that their initial convalescence plan is no longer applicable and that the adjusted instructions of the surgeon should be followed.

###### Getting Feedback on Recovery by a Recovery Monitor and Recovery Report

**Figure 4:**
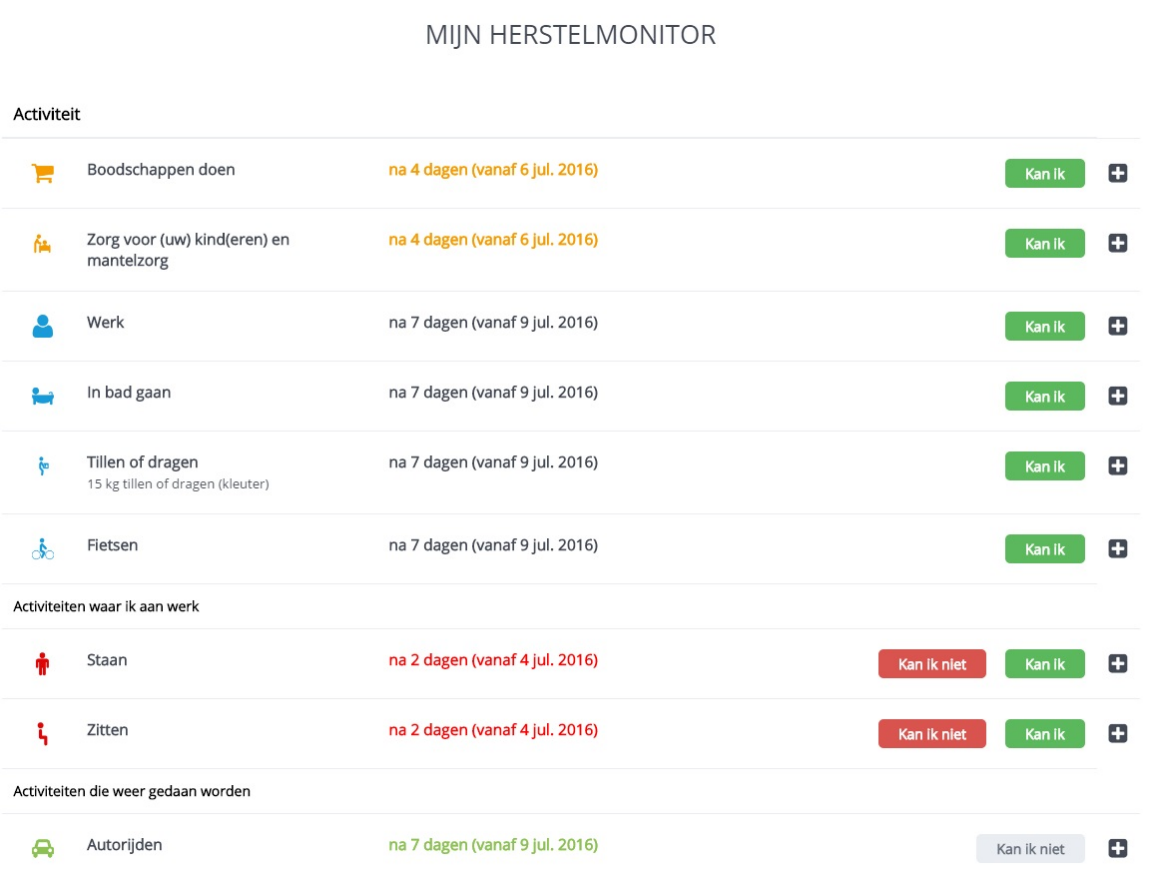
Recovery monitor (see Multimedia Appendix 1 for more information).

**Figure 5:**
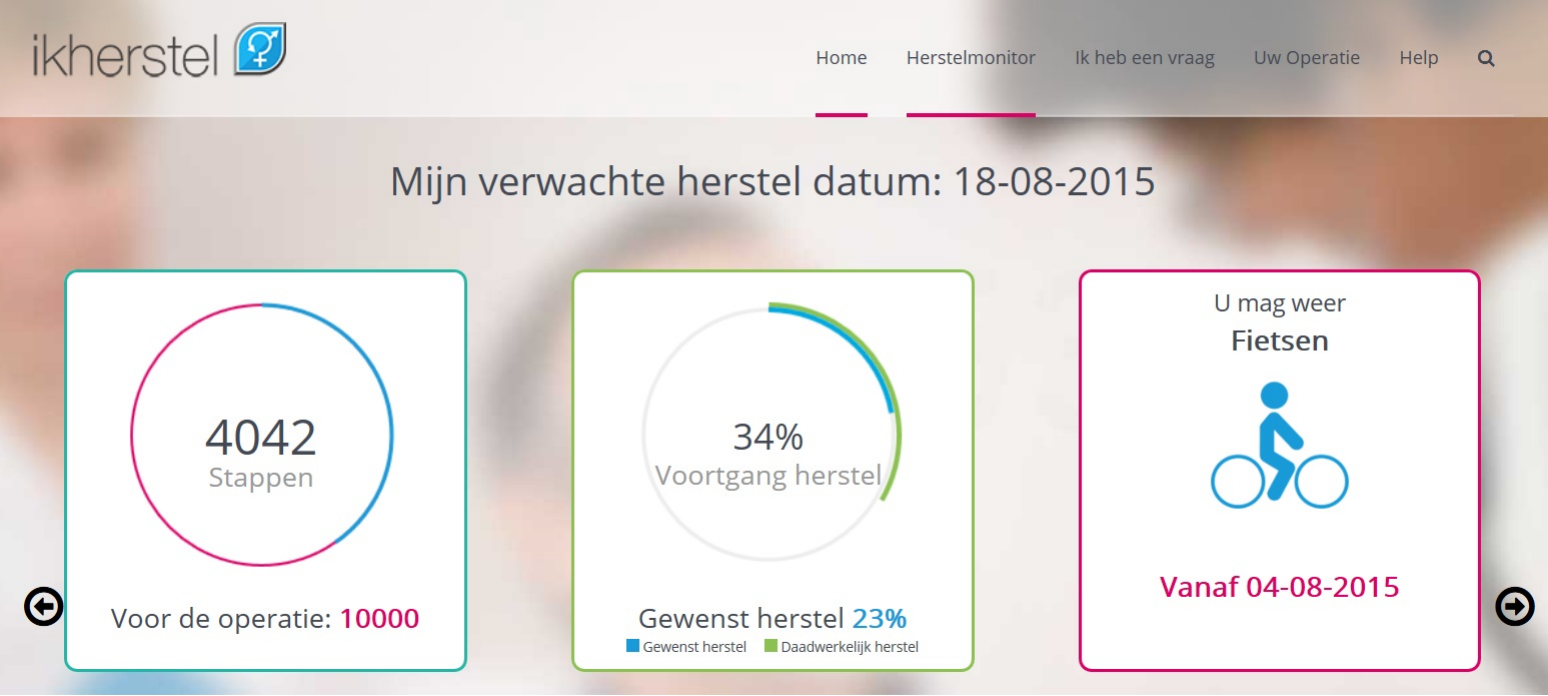
Recovery report (see Multimedia Appendix 1 for more information).

The recovery monitor and report are tools to identify recovery problems and give patients feedback on their recovery progress. Patients are asked to indicate by a recovery monitor to what extent they have resumed their activities (Figure 4), which is subsequently graphically displayed in a recovery report allowing them to track their progress (Figure 5). If patients report a delayed recovery, an alerting system advises them to contact a specific health care professional, depending on the underlying problem. It also aims to improve monitoring and transition of postoperative care; after the patient has given consent, the Web portal can be accessed by a health care provider in secondary care to monitor the patients’ recovery and thus identify recovery problems.

###### Postoperative Consult by eConsultation Instead of Consultation in the Outpatient Clinic

**Figure 6:**
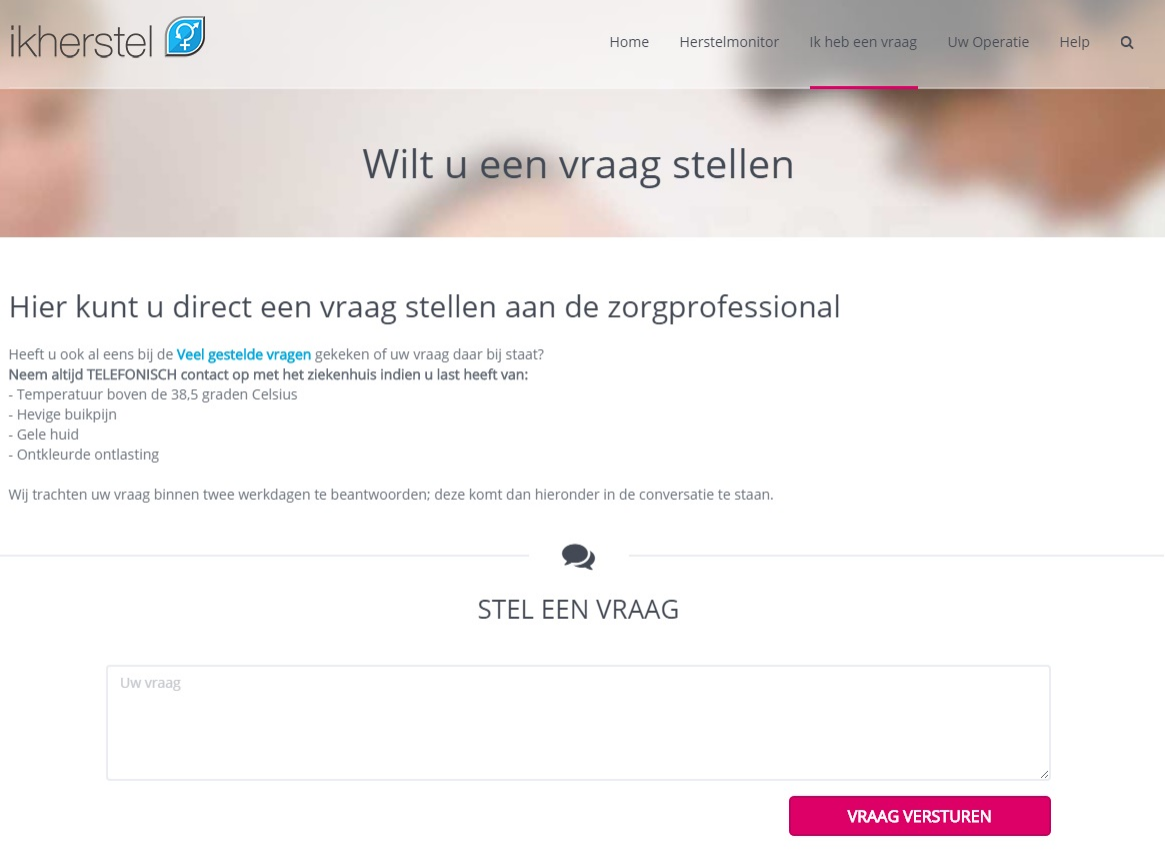
eConsult (see Multimedia Appendix 1 for more information).

At discharge, patients from the intervention group will not receive a standard appointment at the outpatient clinic. Instead, they are offered continuous guidance via the website (information, feedback on recovery) and the possibility to ask questions on the website to a health care professional from their own hospital by means of an eConsult in case of recovery problems (Figure 6). They are informed that the question will be answered within 2 working days except for urgent matters. In that case, they receive a phone number for direct contact. In addition patients will receive a telephone call 2 weeks after surgery to inform them about any test results. Various studies already have proven that telephone follow-up is feasible and effective after the type of surgical procedures which are included in this study [26-28]. The telehealth follow-up aims to replace the standard single postoperative consultation in the outpatient clinic to increase access to care, reduce patient uncertainties and fear related to the recovery process, reduce costs and workload, and meet patient preferences of care during out-of-office hours [29,30]. We hypothesize that patients will be more comfortable and less hampered in resuming their activities with the opportunity to ask questions whenever they prefer instead of the standard consultation several weeks after surgery.

##### Mobile Phone App (mHealth) and Activity Tracker

**Figure 7:**
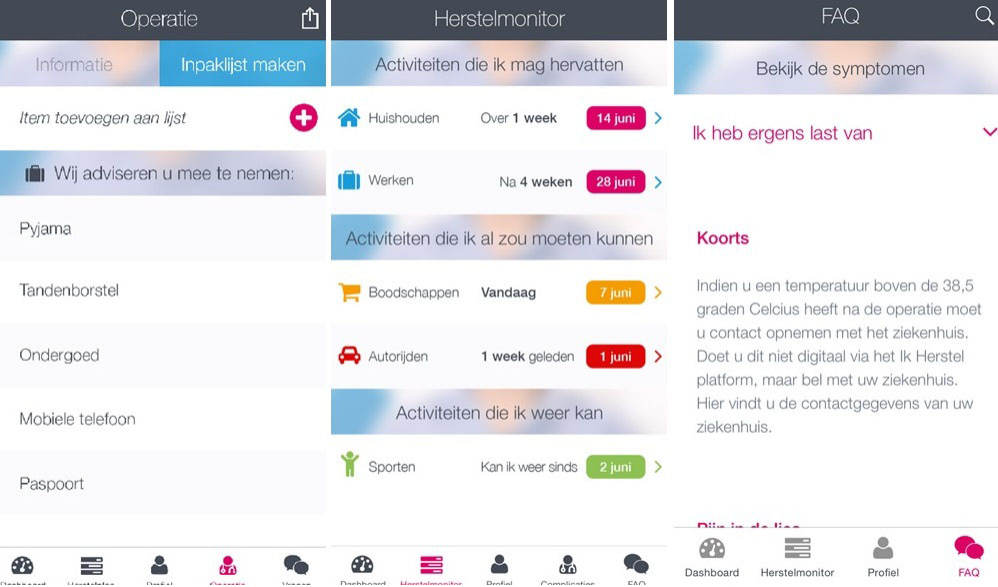
Mobile phone app (see Multimedia Appendix 1 for more information).

**Figure 8:**
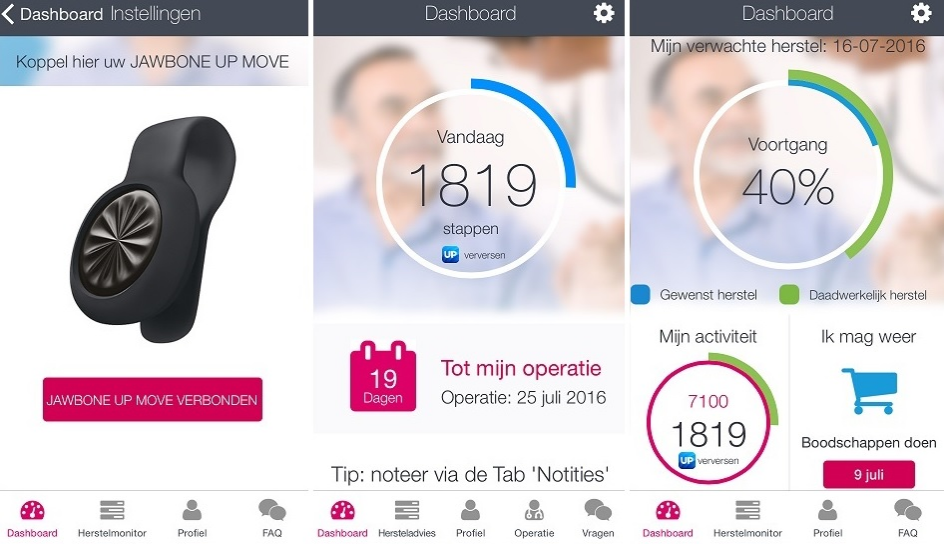
Activity tracker connected to the mobile phone app (see Multimedia Appendix 1 for more information).

All information which is available on the website is also available on the mobile phone app (Ikherstel app), which will be synchronized with the website. This mHealth app has been developed because of the increasing use of mobile phone apps and in order to make the intervention more accessible. This means that the convalescence plan that is created by the patient on the website will also be displayed on the app. In addition, the app will offer some extra features, such as a section to make notes and the option to compose a list of what to pack when being admitted to hospital (Figure 7). If patients do not have a smartphone they will only use the website. Patients who have a smartphone may also receive an activity tracker (UP MOVE, Jawbone). Not all kinds of smartphones can be linked to this activity tracker, so only a proportion of the patients will receive an activity tracker. This activity tracker measures daily step count. We performed a pilot study with an accelerometer in 30 patients who underwent surgery that showed that the step count had a clear correlation with activity intensity levels (data not yet published, personal communication). Patients can link the activity tracker to the app. The activity tracker will be used as an aid for patients to monitor and to give feedback on their recovery. Patients are instructed to wear the accelerometer from the seventh day before surgery until 3 weeks after surgery and during the sixth week after surgery. In the week before surgery a baseline measurement will be performed. The mean step count per day of this week will be set as their target postoperative activity level. In the app, it will be displayed on which date this target level is expected to be reached (Figure 8). This date is based on the convalesce plan which is developed by the patient. After surgery, the daily step count will be graphically displayed in the app and on the website as a percentage of their target activity level. When no baseline measurement is performed (for example, because the period between inclusion and surgery is shorter than 1 week), the target activity level will be set at 7100 steps per day (based on the mean preoperative value of the pilot study). As the pilot study also indicated that the most improvement in activity level was detected in the first 2 weeks after surgery, it was decided to ask the patients to wear the accelerometer during the first 3 weeks after surgery. The sixth week was chosen because we hypothesize that baseline activity level will be reached in this week. Patients will be offered the opportunity to wear the activity tracker in the fourth and fifth week after surgery as well.

### Outcomes

Table 2 presents all outcome measures, the measurement instruments that will be used, and the time points when they will be assessed.

#### Primary Outcome

The primary outcome measure of the study will be time to return to normal activities (RNA). The Patient-Reported Outcomes Measurement Information System (PROMIS) Physical Functioning item bank version 1.2 will be used to measure limitations in daily activities. This item response theory (IRT)–based item bank has been developed and validated in the United States and translated into Dutch-Flemish [31,32]. Initial validation studies in Dutch patients confirmed the unidimensionality and underlying calibration of the IRT model (personal communication, article submitted). This item bank consists of 121 activities. We made a selection of the 29 most relevant activities for this study (Multimedia Appendix 2). We considered the 92 excluded activities as not being affected by the type of surgery (eg, “Can you brush your hair?”) or as duplicate activities. The list of 29 activities will be presented to the patients at baseline (prior to surgery), and they will be asked to select 8 activities which are most relevant for them in daily life. In this way, patients will design their own personal physical functioning short form. Since items in an IRT-based item bank are calibrated onto the same continuum, one can select any subset of questions in that bank and the scores obtained from the derived short forms are comparable to that from the complete bank [33]. In the follow-up questionnaires, patients will only receive those PROMIS questions regarding the 8 activities that they selected before surgery as relevant to their personal life. Items are scored on a 5-point scale with 1 as most limited and 5 as no limitation. In addition, they will be asked postoperatively whether they can perform this activity on the same level as before surgery. If they answer “yes,” they are asked to fill in since when (date), and the question will no longer be repeated in the following questionnaires. If the question is answered with a “no,” the question will be asked again at the next follow-up moment. For each patient, this will result in 8 dates of resumption of their activities. These dates will be converted into time periods by calculating the number of days that have elapsed between the date of surgery and the date of the resumption of that particular activity. The moment of resumption of the last activity on the list will be defined as the RNA moment and thus the primary outcome measure will be time elapsed between surgery and resumption of the last activity. If patients have not resumed 1 or more activities at 6 months, those activities will be censored. If information about the resumption of 2 or fewer activities is missing, the RNA moment will be calculated based on 6 activities. If information about the resumption of more than 2 activities is missing, the RNA moment cannot be calculated accurately and will be considered as missing data.

#### Secondary Outcomes

As secondary outcomes we will use the time until the first activity can be resumed and the moment that 75% of the activities are resumed. Scores of the physical functioning short forms will also be calculated by summarizing for each patient the scores of the 8 activities and transforming them into a T-score on the PROMIS physical functioning metric, where 50 represents the average score of the US population with an SD of 10. The following outcomes will also be measured:

- Social participation: assessed with the PROMIS Ability to Participate in Social Roles and Activities version 2.0 short form 8a [34]
- Self-rated health: measured by the 3-level EuroQol-5D (EQ-5D-3L) [35]
- Durations of return to work (RTW) (only for the working population): the time until the first day on which work will be resumed and the time until full resumption of work activities will be assessed
- Physical activity: assessed by the International Physical Activity Questionnaire (IPAQ) short form [36]
- Length of recovery: measured by the recovery specific quality of life questionnaire (RI-5) short form [37].
- Pain intensity: measured by the Von Korff questionnaire visual analog scale (VAS) [38].
- Patient satisfaction: measured with a self-developed patient satisfaction questionnaire focused on satisfaction with perioperative care, the care program (both groups), the website (both groups), the app (intervention group only), and the activity tracker (intervention group only).

**Table 2:** Outcome measures, measurement instruments, and time points.

				Enrollment	Surgery	Post-allocation				
Time Point^a^				*T*_0_		*T*_1_	*T*_2_	*T*_3_	*T*_4_	*T*_5_
**Enrollment**										
		Eligibility screen		X					
		Informed consent		X					
		Allocation		X					
**Interventions**									
		**Intervention group**							
			Intervention part of the website	XXXXXXXXXXXXXXXXXXXXXXXXXXXXX						
			Mobile phone app	XXXXXXXXXXXXXXXXXXXXXXXXXXXXX						
			Activity tracker	XXXXXXXXXXXXXXXXXXXXXXXXXXXXX						
		**Control group**								
			Nonintervention part of the website	XXXXXXXXXXXXXXXXXXXXXXXXXXXXX							
**Assessments**										
	**Primary outcome measure**								
		Return to normal activities (PROMIS^b^ physical functioning item bank)		X		X	X	X	X	X
	**Secondary outcome measures**									
		Participation (PROMIS short form Social Roles)		X		X	X	X		X
		Self-rated health (EuroQol-5D-3L)		X			X	X	X	X
		Return to work (Return to work questionnaire)				X	X	X	X	X
		Physical activity (IPAQ^c^)		X			X	X	X	
		Recovery (RI-5^d^)		X		X	X	X		
		Pain intensity (VAS^e^)				X	X	X		
		Patient satisfaction (Satisfaction questionnaire)							X	
	**Prognostic factors**									
		Sociodemographic data (Sociodemographic questionnaire)		X						
	**Potential confounding factors**									
		Complications during surgery (Surgical report)			X^f^					
		Postoperative complications (Postoperative medical notes)						X^f^				
	**Process measures**									
		Protocol adherence (Adherence questionnaire)							X	
	**Cost measures**									
		Care program (Bottom-up approach)		X^f^						
		Health care utilization (Cost questionnaire)							X	X
		Informal care (Cost questionnaire)							X	X
		**Productivity loss**								
			Presenteeism: iPCQ^g^ and WHO-HPQ^h^	X		X	X	X	X	X
			Absenteeism: iPCQ	X		X	X	X	X	X
			Unpaid productivity: Cost questionnaire						X	X

^a^T_0_: 1 month before surgery; T_1_: 1 week after surgery; T_2_: 3 weeks after surgery; T_3_: 6 weeks after surgery; T_4_: 3 months after surgery; T_5_: 6 months after surgery.

^b^PROMIS: Patient-Reported Outcomes Measurement Information System.

^c^IPAQ: International Physical Activity Questionnaire.

^d^RI-5: recovery specific quality of life questionnaire short form.

^e^VAS: visual analog scale.

^f^Measured by the research team.

^g^iPCQ: Institute for Medical Technology Assessment (iMTA) Productivity Cost Questionnaire.

^h^WHO-HPQ: World Health Organization—Health and Work Performance Questionnaire.

#### Prognostic Factors

Before surgery (T_0_), various sociodemographic data will be assessed (sex, age, level of education, living conditions, working conditions) and questions asked regarding expectations about the length of recovery. Also the T-scores of the PROMIS physical functioning short forms conducted by the patient will be calculated.

#### Potential Confounding Factors

Major complications during surgery (eg, conversion to an open procedure), major complications in the postoperative course (eg, leading to a prolonged hospital stay of more than 2 nights after surgery), and readmission to the hospital in the 30 days after surgery or repeated surgery in the 30 days after surgery are considered as potential confounders. The complications will be assessed by reviewing the surgical reports and postoperative notes.

#### Process Measures

A process evaluation of the intervention will be carried out in accordance with the Linnan and Steckler method [39]. Table 3 contains the different components of the intervention and the level at which they are measured and in what way.

**Table 3:** Process evaluation.

	Website	App	eConsult	Telephone consult after 2 weeks	Activity tracker
Reach: The proportion of intended target audience that participated in the study	Patients who met the inclusion criteria, signed informed consent, and are randomized to the intervention or control group^a^						
Dose delivered: The number or amount of intended units of each component delivered or provided to the intervention group	Proportion of the patients of the intervention group who received an account for the web portal and app^a^			Proportion of the patients of the intervention group who received a telephonic appointment at discharge^b^	Proportion of the patients of the intervention group who received an activity tracker^a^
Dose received: The extent to which participants from the intervention group actively engage with, interact with, are receptive to, or use materials or recommend resources	Proportion of the patients of the intervention group that made a convalescence plan^c^	Proportion of the patients of the intervention group that used the app^d^	Proportion of the patients of the intervention group that asked one or more questions on the web portal^c^	Proportion of the patients of the intervention group that received their telephonic appointment^b^	Proportion of the patients of the intervention group that connected the activity tracker to their phone^c^
Fidelty: The extent to which the intervention was delivered as planned	Proportion of the convalescence plans that are electronically approved by the specialist^c^	X	Proportion of the questions that were answered^c^	Proportion of the patients of the intervention group that came back at the outpatient office in addition to their telephonic consult^b^	Proportion of the patients of the intervention group that used the activity tracker in the first 3 weeks after surgery (minimum 3 days per week) and in the sixth week after surgery (minimum 3 days)^c^
Participant attitudes: Satisfaction and usage barriers of the intervention	Assessment of the website by the intervention group and reasons for not using the website^d^	Assessment of the app and reasons for not using the app^d^	X	X	Assessment of the activity tracker and reasons for not using the activity tracker^d^

^a^Data collection method: logistic database.

^b^Data collection method: notes in the medical record.

^c^Data collection method: web log.

^d^Data collection method: adherence and satisfaction questionnaire.

#### Cost Measures

##### Identification of Costs

Costs will be measured from a societal and a health care perspective. Societal costs will consist of costs of the intervention (ie, the substitution of perioperative care by eHealth), other health care use, informal care, absenteeism (ie, absence from work), presenteeism (ie, reduced productivity while at work), and unpaid productivity (ie, inability to perform educational activities, chores, volunteer work). When the health care perspective is applied, only costs accruing to the formal health care sector will be included (ie, costs of the intervention and other health care use).

##### Measurement and Valuation of Costs

Intervention costs will include all costs related to the development and implementation of the intervention and will be measured using a bottom-up microcosting approach (ie, detailed data will be collected regarding the quantity of resources consumed during the development and implementation of the intervention as well as their unit prices). All other cost categories will be measured using Web-based questionnaires administered at baseline and after 1 week, 3 weeks, 6 weeks, 3 months, and 6 months of follow-up (Table 2). Health care costs will include costs related to the use of primary care (eg, general practitioner), secondary care (eg, hospital visits), and medication. These costs will be valued using Dutch standard costs and, if unavailable, prices according to professional organizations [40]. Informal care will be valued using a recommended Dutch shadow price [40]. Absenteeism will be measured using the Institute for Medical Technology Assessment (iMTA) Productivity Cost Questionnaire (iPCQ) and valued in accordance with the friction cost approach using the estimated cost of productivity losses in the Netherlands [40,41]. Presenteeism costs will be measured using the iPCQ questionnaire and the World Health Organization Health and Work Performance Questionnaire and valued using the estimated cost of productivity losses in the Netherlands as well [42]. Unpaid productivity costs will be valued using the aforementioned recommended Dutch shadow price [40].

### Sample Size Calculation

Previous studies evaluating this type of intervention on RNA are lacking. We based our sample size calculation on the outcomes on RTW after gynecological surgery of our previous study [16]. Based on this study we expected a hazard ratio (HR) of 1.4 for RNA using the optimized intervention. Considering an HR of 1.4 and using a 2-sided log-rank test at a significance level of 5%, we need to observe 285 events (patients returning to normal activities) to achieve a power of 80%. The total sample size is set at 308 (154 per arm) to account for an anticipated proportion of 2.5% of patients not returning to daily activities within the 6-month follow-up period and a dropout rate of 5%.

### Recruitment and Inclusion

All patients between 18 and 75 years old who are on the waiting list for a laparoscopic cholecystectomy, inguinal hernia surgery, or adnexal surgery in one of the participating hospitals who meet the inclusion criteria will receive a letter of information about the study on behalf of their doctor. After 1 week, contact will be made by phone to evaluate their willingness to participate and to access eligibility. If the patient wants to and is eligible to participate, informed consent will be signed. Participants will not receive any financial or nonfinancial incentives.

### Allocation

After inclusion, patients will be asked to complete the first online questionnaire (T_0_) within the month before surgery. After patients have completed the questionnaire, randomization will take place by means of a computer-based randomization list stratified regarding hospital, sex, and surgical procedure using permuted blocks, size 2. Patients are randomized to the intervention or the control group in a 1:1 ratio. The researcher performing randomization is independent from the recruitment or data analyses.

### Blinding

Patients are blinded to the intervention as they do not know which program is developed as a nonintervention or intervention care program. After allocation, patients will receive an email containing a link to the care program to which they are allocated. Both care programs can be accessed through the website, but after signing in, patients will receive access only to the part of the website to which they are randomized. The researchers involved in the analyses will be blinded to the allocation throughout the analyses. Health care providers cannot be blinded to the intervention because it is highly likely that they will be notified of the allocation either by the patient or the patient’s medical file.

### Data Collection

Data will be collected by means of self-reported electronic questionnaires at standard moments. Data will be collected in the month before surgery (T_0_) and 1 week (T_1_), 3 weeks (T_2_), 6 weeks (T_3_), 3 months (T_4_) and 6 months (T_5_) after surgery (Table 2). When data regarding RNA are missing during 2 measuring moments, an attempt will be made to collect the missing questions by telephone or email.

### Data Analyses

#### Effect Analyses

All analyses will be performed in SPSS (IBM Corp). Baseline characteristics will be summarized using descriptive statistics and compared between the intervention and control group using *t* tests, Mann-Whitney U tests, chi-square tests, or Fisher exact tests. Survival analysis will be used to analyze time until RNA data. Both crude and adjusted analyses will be performed. Hospital, surgical procedure, and sex will be taken into account as covariates in the adjusted analyses because these are the factors for which stratification will apply. In addition, when there are clinically relevant differences between the intervention and the control group in the baseline characteristics or potential confounding factors, this will also be considered as a covariate in further analyses. To describe the distribution of the duration until RNA in both groups, the Kaplan-Meier method will be used. The Cox proportional hazard model will be applied to calculate HRs. Differences in secondary outcomes between the groups will be assessed by mixed models and multilevel logistic regression models for outcomes that are measured more than once during follow-up, and *t* tests, Mann-Whitney U tests, chi-square tests, or Fisher exact tests will be used when differences on 1 time point will be compared. Statistical analyses will be performed according to intention-to-treat principle, which will be compared to per-protocol analyses. Patients will be included in the per-protocol analyses when they used the intervention as intended, which will be defined as the generation of a convalescence plan on the website. This will be measured by a web log. Subgroup analyses will be performed regarding the surgical procedure (1. Cholecystectomy, 2. Hernia inguinal surgery and 3. Adnexal surgery) and type of surgery (gynecological vs surgical procedures). A post hoc analysis will be carried out on patients without major complications (definition described in Potential Confounding Factors).

#### Economic Evaluation

The economic evaluation will be performed from a societal and health care perspective. The time horizon of the economic evaluation is 6 months, thus discounting of costs and effects is not necessary. Both cost-effectiveness and cost-utility analyses will be performed. The cost-effectiveness analysis will be performed with the primary effect measure (ie, RNA). The cost-utility analysis will be performed with quality-adjusted life-years (QALYs). The patients’ EQ-5D-3L health states will be converted into utilities using the Dutch tariff, and QALYs will be calculated using linear interpolation between measurement points [43]. The analyses will be done according to the intention-to-treat principle. Missing cost and effect data will be imputed using multiple imputation after which results will be pooled using Rubin’s rules [44]. Cost and effect differences will be analyzed using multilevel analyses with a 2-level structure (ie, patient, hospital). The 95% CIs around the cost differences will be estimated using bias-corrected intervals with 5000 replications. The bootstrap replications will be stratified for hospital to account for the clustering of data [45]. Incremental cost-effectiveness ratios (ICERs) will be calculated by dividing the difference in mean total costs between the groups by the difference in mean effects. Uncertainty surrounding the ICERs will be graphically presented on cost-effectiveness planes. Cost-effectiveness acceptability curves will also be estimated illustrating the probability that the optimization and substitution of perioperative care by eHealth is cost effective in comparison with usual care for a range of different ceiling ratios. Various sensitivity analyses will be performed to assess the robustness of the results.

## Results

The inclusion process started in September 2015. The expected end date is August 2016. The data collection process will last until February 2017 since the follow-up duration is 6 months. The results are expected in 2018. Data cleaning or analyses have not begun as of this article’s submission.

## Discussion

### Summary

In this RCT we will evaluate the effect of eHealth on RNA after abdominal surgery.

### Strengths and Limitations

In the earlier studies performed by our research group, the effect of the eHealth intervention was evaluated in terms of duration until RTW [16,46]. The reason the primary outcome measure in this study is changed to duration until RNA is that in the earlier studies only employed patients could participate; we believe that unemployed patients can also benefit from this eHealth care program to facilitate recovery. However, one of the reasons for chosing duration until RTW in the earlier performed studies was that this outcome measure was relatively easy to measure and objectify. Duration until RNA is more difficult to measure and objectify. Most studies using this outcome measure make use of fixed measurement instruments, which may contain questions not always applicable to all patients or may not represent the most relevant problems for patients. As far as we know, this is the first study in which RNA after surgery is measured by a measurement instrument tailored to the patient. A tailored instrument increases the validity and reliability of the primary outcome measure. The ultimate form of tailoring is computer-adaptive testing (CAT), in which after the first item, the selection of items is determined by the person’s responses to previous items. For example, Zanocco et al [47] used CAT to measure changes in patient-reported health before and after parathyroidectomy. PROMIS short forms and CATs are increasingly used as outcome measures across clinical studies in different fields [48]. In our study we will use individualized short forms because CAT software for use in the Netherlands is still under development [49]. Another strength of this study is that we developed a nonintervention (standard) website, which enables us to blind the patients to which group they are assigned. Blinding of the health care professionals, however, was not possible. But in our opinion this will not influence the study results, since the health care professionals do not play a substantial role in the intervention or of the data-collecting process. This study is also strengthened by the fact that state-of-the-art statistical methods, such as multiple imputation, bootstrapping, and multilevel analyses, will be used. A limitation of the study is the heterogeneity of surgical procedures. However, the Delphi study that we performed to compose the convalescence recommendations used in this study showed more or less the same convalescence recommendations for all surgical procedures, which suggests that the recovery periods of these surgical procedures are comparable [24,25]. In addition, the heterogeneity can be considered an advantage, because the results will be applicable to a broader population. Another limitation may be that the study is carried out in 7 hospitals, which means that the usual care that the control group receives can vary between hospitals. We tried to reduce this bias by stratifying the randomization by hospital. Finally, the baseline measurement with the activity tracker will be performed in the week before surgery and therefore cannot assure that the baseline measurement will give a representative view of the normal activity pattern of the patients. However, the patients who participate in the study will undergo elective surgery. This in general will mean that the patients will not be suffering from an acute illness or bedridden at the time of surgery. Although we cannot entirely rule out that the baseline measurement will be influenced, we nevertheless believe that the risk will be minimal.

### Comparison to Prior Work: What This Study Will Add

To our knowledge, there are 13 other studies which have evaluated an educational or supportive eHealth intervention in perioperative care [16,46,50-61]. Most of these studies were relatively small or did not perform a power calculation. Only 2 studies were carried out in patients undergoing abdominal surgery. In addition, almost all studies (12/13) aimed to evaluate the eHealth intervention in addition to usual care. Only 1 study aimed to evaluate eHealth as (partial) substitution of usual care; however, this study had no report of a power calculation and included patients undergoing orthopedic surgery. The most comparable study to ours is that of Bouwsma et al [46], which evaluated the cost-effectiveness of an eHealth intervention in patients undergoing gynecological surgery (results not yet published). The main differences with our study are the primary outcome measure (RNA vs RTW), the aim of the eHealth intervention (substitution of care vs additional care), and the patient population (working and nonworking men and women vs working women). A total of 9 studies found a significant positive effect of the eHealth intervention regarding an outcome measure focusing on the postoperative course. However, studies evaluating eHealth interventions in abdominal surgery to substitute usual care are lacking, which underlines the importance of this current study.

### Clinical Relevance

Improving the quality of perioperative care is required because postoperative care is limited due to the reduction in the length of hospital stay. In addition, because of the increasing demand for health care due to the aging population and personnel shortages in health care, it is necessary to deliver more efficient and cost effective perioperative care. This study will evaluate whether eHealth can be used to suit this purpose. It will both give insight for health care professionals by determining the best form of perioperative care and facilitate policy makers in deciding whether eHealth can be used to substitute usual care against lower costs. The generalizability of this study is high because the eHealth intervention will be evaluated in various types of surgical procedures and will only require minor adaptions in order to be applied to other types of procedures.

## Appendix

Multimedia Appendix 1 Further information on screenshots.

Multimedia Appendix 2 Selection of the Patient-Reported Outcomes Measurement Information System (PROMIS) physical functioning item bank version 1.2.

Multimedia Appendix 3 Peer review report 1.

Multimedia Appendix 4 Peer review report 2.

Multimedia Appendix 5 Peer review report 3.

## Abbreviations

CAT: computer-adaptive testing
eHealth: electronic health
EQ-5D-3L: EuroQol-5D (3 level)
HR: hazard ratio
ICER: incremental cost-effectiveness ratio
IPAQ: International Physical Activity Questionnaire
iPCQ: Institute for Medical Technology Assessment (iMTA) Productivity Cost Questionnaire
IRT: item response theory
mHealth: mobile health
PROMIS: Patient-Reported Outcomes Measurement Information System
QALY: quality-adjusted life-years
RCT: randomized controlled trial
RI-5: recovery specific quality of life questionnaire short form
RNA: return to normal activities
RTW: return to work
VAS: visual analog scale
WHO-HPQ: World Health Organization Health and Work Performance Questionnaire

## References

[1] American Hospital Association Chartbook: Trends Affecting Hospitals and Health Systems.

[2] Bachoo P, Duncan J (1995). Prolonged convalescence following inguinal hernia repair: an unnecessary trend. Health Bull (Edinb).

[3] Baker D, Rider M, Fawcett A (1994). When to return to work following a routine inguinal hernia repair: Are doctors giving the correct advice?. J R Coll Surg Edinb.

[4] Clayton M, Verow P (2007). Advice given to patients about return to work and driving following surgery. Occup Med (Lond).

[5] Ottesen M, Møller C, Kehlet H, Ottesen B (2001). Substantial variability in postoperative treatment, and convalescence recommendations following vaginal repair: a nationwide questionnaire study. Acta Obstet Gynecol Scand.

[6] Bisgaard T, Klarskov B, Rosenberg J, Kehlet H (2001). Factors determining convalescence after uncomplicated laparoscopic cholecystectomy. Arch Surg.

[7] Callesen T, Klarskov B, Bech K, Kehlet H (1999). Short convalescence after inguinal herniorrhaphy with standardised recommendations: duration and reasons for delayed return to work. Eur J Surg.

[8] Brölmann HAM, Vonk Noordegraaf A, Bruinvels DJ, de Vet RH, Dirksz AA, Huirne JAF (2009). Can prolonged sick leave after gynecologic surgery be predicted? An observational study in The Netherlands. Surg Endosc.

[9] Johansen P, Al-Khafagi SK, Thostesen LM, Lauszus FF, Rasmussen KL (2008). Analysis of need for sick leave after hysterectomy. Ugeskr Laeger.

[10] Henderson M, Glozier N, Holland EK (2005). Long term sickness absence. BMJ.

[11] Waddell G, Burton K, Aylward M (2007). Work and common health problems. J Insur Med.

[12] Barello S, Triberti S, Graffigna G, Libreri C, Serino S, Hibbard J, Riva G (2015). eHealth for Patient Engagement: a systematic review. Front Psychol.

[13] Tang C, Lorenzi N, Harle CA, Zhou X, Chen Y (2016). Interactive systems for patient-centered care to enhance patient engagement. J Am Med Inform Assoc.

[14] Vonk Noordegraaf A, Huirne JAF, Pittens CA, van Mechelen W, Broerse JE, Brölmann HA, Anema JR (2012). eHealth program to empower patients in returning to normal activities and work after gynecological surgery: intervention mapping as a useful method for development. J Med Internet Res.

[15] Pittens CACM, Vonk Noordegraaf A, van Veen SC, Anema J, Huirne JAF, Broerse JEW (2015). The involvement of gynaecological patients in the development of a clinical guideline for resumption of (work) activities in the Netherlands. Health Expect.

[16] Vonk Noordegraaf A, Anema JR, van Mechelen W, Knol DL, van Baal WM, van Kesteren PJM, Brölmann HAM, Huirne JAF (2014). A personalised eHealth programme reduces the duration until return to work after gynaecological surgery: results of a multicentre randomised trial. BJOG.

[17] Wantland D, Portillo C, Holzemer W, Slaughter R, McGhee E (2004). The effectiveness of Web-based vs non-Web-based interventions: a meta-analysis of behavioral change outcomes. J Med Internet Res.

[18] Webb T, Joseph J, Yardley L, Michie S (2010). Using the Internet to promote health behavior change: a systematic review and meta-analysis of the impact of theoretical basis, use of behavior change techniques, and mode of delivery on efficacy. J Med Internet Res.

[19] Chan A, Tetzlaff J, Gøtzsche PC, Altman D, Mann H, Berlin J, Dickersin K, Hróbjartsson A, Schulz K, Parulekar W, Krleza-Jeric K, Laupacis A, Moher D (2013). SPIRIT 2013 explanation and elaboration: guidance for protocols of clinical trials. BMJ.

[20] Moher D, Hopewell S, Schulz K, Montori V, Gøtzsche PC, Devereaux P, Elbourne D, Egger M, Altman D (2010). CONSORT 2010 explanation and elaboration: updated guidelines for reporting parallel group randomised trials. BMJ.

[21] Eysenbach G (2011). CONSORT-EHEALTH: improving and standardizing evaluation reports of Web-based and mobile health interventions. J Med Internet Res.

[22] Yin B, Goldsmith L, Gambardella R (2015). Web-based education prior to knee arthroscopy enhances informed consent and patient knowledge recall: a prospective, randomized controlled study. J Bone Joint Surg Am.

[23] Vonk Noordegraaf A, Anema J, Louwerse M, Heymans M, van M, Brölmann HAM, Huirne JAF (2014). Prediction of time to return to work after gynaecological surgery: a prospective cohort study in the Netherlands. BJOG.

[24] van Vliet DCR, van der Meij E, Bouwsma EVA, Vonk Noordegraaf N, van den Heuvel B, Meijerink WJHJ, van Baal WM, Huirne JAF, Anema J (2016). A modified Delphi method toward multidisciplinary consensus on functional convalescence recommendations after abdominal surgery. Surg Endosc.

[25] Huirne JAF, Brölmann HAM, van Mechelen W, Anema J, Vonk Noordegraaf A (2011). Multidisciplinary convalescence recommendations after gynaecological surgery: a modified Delphi method among experts. BJOG.

[26] Eisenberg D, Hwa K, Wren SM (2015). Telephone follow-up by a midlevel provider after laparoscopic inguinal hernia repair instead of face-to-face clinic visit. JSLS.

[27] Hwa K, Wren SM (2013). Telehealth follow-up in lieu of postoperative clinic visit for ambulatory surgery: results of a pilot program. JAMA Surg.

[28] Chen DW, Davis RW, Balentine CJ, Scott AR, Gao Y, Tapia NM, Berger DH, Suliburk JW (2014). Utility of routine postoperative visit after appendectomy and cholecystectomy with evaluation of mobile technology access in an urban safety net population. J Surg Res.

[29] Armstrong KA, Semple JL, Coyte PC (2014). Replacing ambulatory surgical follow-up visits with mobile app home monitoring: modeling cost-effective scenarios. J Med Internet Res.

[30] Vimalananda V, Gupte G, Seraj S, Orlander J, Berlowitz D, Fincke B, Simon S (2015). Electronic consultations (e-consults) to improve access to specialty care: a systematic review and narrative synthesis. J Telemed Telecare.

[31] Rose M, Bjorner JB, Gandek B, Bruce B, Fries JF, Ware JE (2014). The PROMIS Physical Function item bank was calibrated to a standardized metric and shown to improve measurement efficiency. J Clin Epidemiol.

[32] Terwee CB, Roorda LD, de Vet HCW, Dekker J, Westhovens R, van Leeuwen J, Cella D, Correia H, Arnold B, Perez B, Boers M (2014). Dutch-Flemish translation of 17 item banks from the patient-reported outcomes measurement information system (PROMIS). Qual Life Res.

[33] Cella D, Gershon R, Lai J, Choi S (2007). The future of outcomes measurement: item banking, tailored short-forms, and computerized adaptive assessment. Qual Life Res.

[34] Bode R, Hahn E, DeVellis R, Cella D, Patient-Reported Outcomes Measurement Information System Social Domain Working Group (2010). Measuring participation: the Patient-Reported Outcomes Measurement Information System experience. Arch Phys Med Rehabil.

[35] Dolan P (1997). Modeling valuations for EuroQol health states. Med Care.

[36] Craig C, Marshall A, Sjöström M, Bauman A, Booth M, Ainsworth B, Pratt M, Ekelund U, Yngve A, Sallis J, Oja P (2003). International physical activity questionnaire: 12-country reliability and validity. Med Sci Sports Exerc.

[37] Kluivers KB, Hendriks JCM, Mol BWJ, Bongers MY, Vierhout ME, Brölmann HAM, de Vet HCW (2008). Clinimetric properties of 3 instruments measuring postoperative recovery in a gynecologic surgical population. Surgery.

[38] Von K, Ormel J, Keefe F, Dworkin S (1992). Grading the severity of chronic pain. Pain.

[39] Linnan L, Steckler A (2002). Process evaluation for public health interventions and research. 1st edition.

[40] https://www.zorginstituutnederland.nl/binaries/content/documents/zinl-www/documenten/publicaties/overige-publicaties/1602-richtlijn-voor-het-uitvoeren-van-economische-evaluaties-in-de-gezondheidszorg-bijlagen/Richtlijn+voor+het+uitvoeren+van+economische+evaluaties+in+de+gezondheidszorg+(verdiepingsmodules).pdf.

[41] Bouwmans C, Krol M, Severens H, Koopmanschap M, Brouwer W, Hakkaart-van RL (2015). The iMTA productivity cost questionnaire: a standardized instrument for measuring and valuing health-related productivity losses. Value Health.

[42] Kessler R, Ames M, Hymel P, Loeppke R, McKenas D, Richling D, Stang P, Ustun T (2004). Using the World Health Organization Health and Work Performance Questionnaire (HPQ) to evaluate the indirect workplace costs of illness. J Occup Environ Med.

[43] Lamers LM, Stalmeier PFM, McDonnell J, Krabbe PFM, van Busschbach JJ (2005). Measuring the quality of life in economic evaluations: the Dutch EQ-5D tariff. Ned Tijdschr Geneeskd.

[44] White IR, Royston P, Wood AM (2011). Multiple imputation using chained equations: Issues and guidance for practice. Stat Med.

[45] Meijer E, Busing FMTA, de Leeuw J, Meijer E (2008). Resampling multilevel models. Handbook of Multilevel Analysis.

[46] Bouwsma EV, Anema JR, Vonk Noordegraaf A, Knol DL, Bosmans JE, Schraffordt Koops SE, van Kesteren PJ, van Baal WM, Lips JP, Emanuel MH, Scholten PC, Mozes A, Adriaanse AH, Brölmann HA, Huirne JA (2014). The cost effectiveness of a tailored, Web-based care program to enhance postoperative recovery in gynecologic patients in comparison with usual care: protocol of a stepped wedge cluster randomized controlled trial. JMIR Res Protoc.

[47] Zanocco K, Butt Z, Kaltman D, Elaraj D, Cella D, Holl JL, Sturgeon C (2015). Improvement in patient-reported physical and mental health after parathyroidectomy for primary hyperparathyroidism. Surgery.

[48] Witter J (2016). The Promise of Patient-Reported Outcomes Measurement Information System—Turning theory into reality: a uniform approach to patient-reported outcomes across rheumatic diseases. Rheum Dis Clin North Am.

[49] Dutch-Flemish PROMIS.

[50] Barnason S, Zimmerman L, Nieveen J, Schmaderer M, Carranza B, Reilly S (2003). Impact of a home communication intervention for coronary artery bypass graft patients with ischemic heart failure on self-efficacy, coronary disease risk factor modification, and functioning. Heart Lung.

[51] Barnason S, Zimmerman L, Nieveen J, Hertzog M (2006). Impact of a telehealth intervention to augment home health care on functional and recovery outcomes of elderly patients undergoing coronary artery bypass grafting. Heart Lung.

[52] Barnason S, Zimmerman L, Nieveen J, Schulz P, Miller C, Hertzog M, Tu C (2009). Influence of a symptom management telehealth intervention on older adults' early recovery outcomes after coronary artery bypass surgery. Heart Lung.

[53] van den Brink JL, Moorman P, de Boer MF, Hop WCJ, Pruyn PFA, Verwoerd CDA, van Bemmel JH (2007). Impact on quality of life of a telemedicine system supporting head and neck cancer patients: a controlled trial during the postoperative period at home. J Am Med Inform Assoc.

[54] Goldsmith DM, Safran C (1999). Using the Web to reduce postoperative pain following ambulatory surgery. Proc AMIA Symp.

[55] Heikkinen K, Leino-Kilpi H, Vahlberg T, Salanterä S (2012). Ambulatory orthopaedic surgery patients’ symptoms with two different patient education methods. International Journal of Orthopaedic and Trauma Nursing.

[56] Martorella G, Côté J, Racine M, Choinière M (2012). Web-based nursing intervention for self-management of pain after cardiac surgery: pilot randomized controlled trial. J Med Internet Res.

[57] Miller C, Zimmerman L, Barnason S, Nieveen J (2007). Impact of an early recovery management intervention on functioning in postoperative coronary artery bypass patients with diabetes. Heart Lung.

[58] Neary P, Sung R, Corrigan M, O'Donovan M, Cahill R, Redmond H (2010). The benefits of an interactive, individualized online patient pathway for patients undergoing minimally invasive radioguided parathyroidectomy: a prospective, double-blinded, randomized clinical trial. Surg Innov.

[59] Yin B, Goldsmith L, Gambardella R (2015). Web-based education prior to knee arthroscopy enhances informed consent and patient knowledge recall: a prospective, randomized controlled study. J Bone Joint Surg Am.

[60] Zimmerman L, Barnason S, Nieveen J, Schmaderer M (2004). Symptom management intervention in elderly coronary artery bypass graft patients. Outcomes Manag.

[61] van der Meij E, Anema J, Otten RHJ, Huirne JAF, Schaafsma F (2016). The effect of perioperative eHealth interventions on the postoperative course: a systematic review of randomised and non-randomised controlled trials. PLoS One.

