# Supplementary material for: Substitution of Usual Perioperative Care by eHealth to Enhance Postoperative Recovery in Patients Undergoing General Surgical or Gynecological Procedures: Study Protocol of a Randomized Controlled Trial
Source: JMIR Res Protoc. 2016 Dec 21;5(4):e245. doi: 10.2196/resprot.6580 (PMC5215129; doi:10.2196/resprot.6580)
Supplement: Multimedia Appendix 1 [file resprot_v5i4e245_app1.pdf]

Appendix 1 - Figure subscripts

Figure 1 - Text and animations on the website
The content is subdivided into three parts (the three pink tabs): Information to prepare patients for surgery (left tab), information about the day of surgery and the surgical procedure (middle tab) and information about the recovery period (right tab).

Figure 2 - Personalized convalescence plan
This is an example of a convalescence plan generated by a patient who will undergo a laparoscopic cholecystectomy. At the top, the date of surgery is displayed. On the left-hand side the activities are displayed which the patient has selected as being relevant for him or her. From top to bottom these include: carrying or lifting (5kg), sitting down for a period of 2 hours, standing upright for a period of 15 minutes after each other, walking for a period of 5-15 minutes, shopping, taking care of someone, working, sports. On the right-hand side the corresponding resumption dates and times are displayed (calculated from the day of surgery).

Figure 3 - Personalized convalescence plan displayed on a time line
Here a convalescence plan is displayed on a timeline, based on the activities selected by the patient who will undergo an open inguinal hernia procedure. These are translated as follows: Day of surgery, standing upright for a period of 15 minutes, sitting down for a period of 2 hours, shopping, taking care of someone, working, driving a car, taking a bath, walking for a period of 1 hour, carrying or lifting of 15 kg, cycling, expected date of full recovery for this patient (based on the selected activities). Shopping, taking care of someone and work are 'composed activities', which means that the patients selected several items to compose the activity. For example, for work he or she selected: lifting of 15kg, 4 hours of standing during the day, 2 hours of sitting during the day, walking for a period of 1 hour, being active in a bent position and a period of work extending across 30 hours per week.

Figure 4 - Recovery monitor
The figure presents the recovery monitor of a patient who underwent a laparoscopic cholecystectomy. The activities which the patient has selected for developing his or her convalescence plan are displayed on the left-hand side. In the middle the corresponding resumption dates are presented and on the right-hand side, the patient can select whether he or she has resumed this activity (by selecting the green button) or not (by selecting the red button). Activities which the patient resumed are then displayed in green under the subheading 'activiteiten die weer gedaan worden', which means 'activities which have been resumed'. Activities which the patient is allowed to resumed but is not able to, are presented in red under the subheading 'activiteiten waar ik aan werk' which means 'activities in progress'. Activities in blue present activities with resumption dates in the future. Activities in orange may be resumed the same day.

Figure 5 - Recovery report
Here a screenshot of the website after surgery is presented. At the top the expected recovery date is displayed. In the left square the results of the activity tracker (described below under subheading 'Mobile phone application (M-health) and an activity tracker') are presented. In the middle square the recovery report is presented. The green line indicates the recovery progress of the patient (based

on the activities which the patient selected as ‘I have resumed’. This is also presented by the corresponding ‘34% recovery progress’. When the patients have selected all the activities as ‘I resumed’, the green line will close the circle and the recovery progress percentage will be 100%. The blue line indicates the recommend recovery based on the recovery advice. The blue line will close the circle on 18 August 2015 (the expected recovery date). In this example you can see that the blue line is longer than the green line, meaning the patient’s recovery progress to be quicker than expected. In the right square, the first activity which the patient may resume and the corresponding resumption date are presented.

Figure 6 - E-consult
In the white area patients can type their personal questions. They can send their question by selecting the pink button. In the text at the top it is explained that the question will be answered within two working days and that the patient has to phone the hospital for urgent matters.

Figure 7 – Mobile phone application
Three screenshots of the app are presented in this figure. The left-hand screenshot presents the packing list. The middle screenshot presents the convalescence plan displayed in the recovery monitor. The right-hand screenshot presents the frequently asked questions (FAQ).

Figure 8 – Activity tracker connected to the mobile phone application
This figure contains three screenshots of the app as well. The screenshot on the left presents the activity tracker which can be connected to the app by selecting the pink button. The screenshot in the middle presents the dashboard of the app before surgery. The circle indicates the number of steps taken that day. In the pink square the number of days until surgery are presented (i.e. 19) and next to this the date of surgery is displayed. The right-hand screenshot contains an image of the app after surgery. The large circle presents the recovery report which is synchronized to the recovery report of the website (for explanations see figure 3B). The small circle indicates the report of the activity tracker. The number in pink (i.e. 7100) presents the mean number of steps taken per day by the patient before surgery, which is the target postoperative activity level. At the top the date is displayed when this target level is expected to be reached. This means that this specific person is expected to reach their target postoperative level of 7100 steps per day on 16 July 2016. The green line indicates the steps taken on that day so far, also presented by the black number (i.e. 1819). On the right-hand side the first activity which the patient may resume and corresponding resumption date are presented.
