# Supplementary material for: Substitution of Usual Perioperative Care by eHealth to Enhance Postoperative Recovery in Patients Undergoing General Surgical or Gynecological Procedures: Study Protocol of a Randomized Controlled Trial
Source: JMIR Res Protoc. 2016 Dec 21;5(4):e245. doi: 10.2196/resprot.6580 (PMC5215129; doi:10.2196/resprot.6580)
Supplement: Multimedia Appendix 2 [file resprot_v5i4e245_app2.pdf]

Appendix 2 – Selection of the PROMIS physical function item bank v.1.2

Question	Answering options
PFB42 Are you able to stand unsupported for 30 minutes?	5 Without any difficulty<br>4 With a little difficulty<br>3 With some difficulty<br>2 With much difficulty<br>1 Unable to do
PFA10 Are you able to stand for one hour?	5 Without any difficulty<br>4 With a little difficulty<br>3 With some difficulty<br>2 With much difficulty<br>1 Unable to do
PFB49 Does your health now limit you in going for a short walk (less than 15 minutes)?	5 Not at all<br>4 Very little<br>3 Somewhat<br>2 Quite a lot<br>1 Cannot do
PFA23 Are you able to go for a walk of at least 15 minutes?	5 Without any difficulty<br>4 With a little difficulty<br>3 With some difficulty<br>2 With much difficulty<br>1 Unable to do
PFB5r1 Does your health now limit you in hiking a couple of miles (3 km) on uneven surfaces, including hills?	5 Not at all<br>4 Very little<br>3 Somewhat<br>2 Quite a lot<br>1 Cannot do
PFB24 Are you able to run a short distance, such as to catch a bus?	5 Without any difficulty<br>4 With a little difficulty<br>3 With some difficulty<br>2 With much difficulty<br>1 Unable to do
PFB13 Are you able to carry a shopping bag or briefcase?.	5 Without any difficulty<br>4 With a little difficulty<br>3 With some difficulty<br>2 With much difficulty<br>1 Unable to do
PFA14r1 Are you able to carry a heavy object (over 10 pounds/5 kg)?	5 Without any difficulty<br>4 With a little difficulty<br>3 With some difficulty<br>2 With much difficulty<br>1 Unable to do
PFB39r1 Are you able to reach and get down a 5 pound (2 kg) object from above your head?	5 Without any difficulty<br>4 With a little difficulty<br>3 With some difficulty<br>2 With much difficulty<br>1 Unable to do
PFC10 Does your health now limit you in climbing one flight of stairs?	5 Not at all<br>4 Very little<br>3 Somewhat<br>2 Quite a lot<br>1 Cannot do
PFC10 Does your health now limit you in climbing several flights of stairs?	5 Not at all<br>4 Very little<br>3 Somewhat<br>2 Quite a lot

	1 Cannot do
PFA3 Does your health now limit you in bending, kneeling, or stooping?	5 Not at all<br>4 Very little<br>3 Somewhat<br>2 Quite a lot<br>1 Cannot do
PFB43 Does your health now limit you in taking care of your personal needs (dress, comb hair, toilet, eat, bathe)?	5 Not at all<br>4 Very little<br>3 Somewhat<br>2 Quite a lot<br>1 Cannot do
PFA53 Are you able to run errands and shop?	5 Without any difficulty<br>4 With a little difficulty<br>3 With some difficulty<br>2 With much difficulty<br>1 Unable to do
PFB11 Are you able to wash dishes, pots, and utensils by hand while standing at a sink?	5 Without any difficulty<br>4 With a little difficulty<br>3 With some difficulty<br>2 With much difficulty<br>1 Unable to do
PFA42 Are you able to carry a laundry basket up a flight of stairs?	5 Without any difficulty<br>4 With a little difficulty<br>3 With some difficulty<br>2 With much difficulty<br>1 Unable to do
PFB12 Are you able to make a bed, including spreading and tucking in bed sheets?	5 Without any difficulty<br>4 With a little difficulty<br>3 With some difficulty<br>2 With much difficulty<br>1 Unable to do
PFB3 Does your health now limit you in putting a trash bag outside?	5 Not at all<br>4 Very little<br>3 Somewhat<br>2 Quite a lot<br>1 Cannot do
PFB1 Does your health now limit you in doing moderate work around the house like vacuuming, sweeping floors or carrying in groceries?	5 Not at all<br>4 Very little<br>3 Somewhat<br>2 Quite a lot<br>1 Cannot do
PFA25 Are you able to do yard work like raking leaves, weeding, or pushing a lawn mower?	5 Without any difficulty<br>4 With a little difficulty<br>3 With some difficulty<br>2 With much difficulty<br>1 Unable to do
PFA4 Does your health now limit you in doing heavy work around the house like scrubbing floors, or lifting or moving heavy furniture?	5 Not at all<br>4 Very little<br>3 Somewhat<br>2 Quite a lot<br>1 Cannot do
PFA33 Are you able to exercise hard for half an hour?	5 Without any difficulty<br>4 With a little difficulty<br>3 With some difficulty<br>2 With much difficulty<br>1 Unable to do

PFA13 Are you able to exercise for an hour?	5 Without any difficulty<br>4 With a little difficulty<br>3 With some difficulty<br>2 With much difficulty<br>1 Unable to do
PFA19r1 Are you able to run or jog for two miles (3km)?	5 Without any difficulty<br>4 With a little difficulty<br>3 With some difficulty<br>2 With much difficulty<br>1 Unable to do
PFC7r1 Are you able to run five miles (8 km)?	5 Without any difficulty<br>4 With a little difficulty<br>3 With some difficulty<br>2 With much difficulty<br>1 Unable to do
PFC33r1 Are you able to run ten miles (16 km)?	5 Without any difficulty<br>4 With a little difficulty<br>3 With some difficulty<br>2 With much difficulty<br>1 Unable to do
PFB7 Does your health now limit you in doing strenuous activities such as backpacking, skiing, playing tennis, bicycling or jogging?	5 Not at all<br>4 Very little<br>3 Somewhat<br>2 Quite a lot<br>1 Cannot do
PFC12 Does your health now limit you in doing two hours of physical labor?	5 Not at all<br>4 Very little<br>3 Somewhat<br>2 Quite a lot<br>1 Cannot do
PFC35 Does your health now limit you in doing eight hours of physical labor?	5 Not at all<br>4 Very little<br>3 Somewhat<br>2 Quite a lot<br>1 Cannot do
