# Supplementary material for: Substitution of Usual Perioperative Care by eHealth to Enhance Postoperative Recovery in Patients Undergoing General Surgical or Gynecological Procedures: Study Protocol of a Randomized Controlled Trial
Source: JMIR Res Protoc. 2016 Dec 21;5(4):e245. doi: 10.2196/resprot.6580 (PMC5215129; doi:10.2196/resprot.6580)
Supplement: Multimedia Appendix 3 [file resprot_v5i4e245_app3.pdf]

Subsidieprogramma / Subsidy programme	:	**DoelmatigheidsOnderzoek 2013-2015**
Dossiernummer / Dossier number	:	**80-83700-98-42074**
Aanvrager / applicant	:	**Prof. dr. J.R. Anema MD PhD**
Projecttitel / Project title	:	**SUBSTITUTION OF USUAL PERIOPERATIVE CARE BY E-HEALTH & ICT: A cost-effectiveness analysis alongside a stepped wedge cluster randomised controlled trial**
Beoordelingscode / Assessment code	:	**B.2013.013C7**

1. Objective and problem definition

Legenda: + (+), +/- (+/-), - (-)

1.1 Objective and problem definition

+	+/-	-
X		

Consider:

- how clear and specific is the objective?;
- how clear and verifiable are the problem definition and hypothesis and is it consistent with the objective?;

Please indicate the strong and weak(er) points.

The introduction to the grant presents a very clearly articulated issue-- rising number of surgeries and greater reliance on one-day hospital stays. A strong case is made for the importance of providing post-operative guidance through a web portal service.

The study is focused on efficiency through a controlled RCT at the hospital level using a step-wise design. The efficiency of health services is an important concern, given the rising number of surgeries, rising health care costs. and move towards one-day hospital stays.

The problem definition is clearly verified with an example provided on hernia care, and a previous study undertaken by the authors on gynecological surgery. In this study the focus will be on abdominal surgery.

There are statements made that could use references to validate them, e.g., "one of the most important reasons [for slow implementation of innovation] is that healthcare innovations are professionally driven" (page 6).

Some minor issues: 1) acronyms are used without defining them-- ICT is never spelled out in full; and 2) there are many sloppy grammatical mistakes through the applications.

2. Strategy

Legenda: + (+), +/- (+/-), - (-)

2.1 Clinical study

+	+/-	-
X		

Consider:

- clarity;
- adequacy in terms of problem definition;
- adequacy of study design, outcome parameters (patient oriented), sample size calculation and analyses;
- relevant differences within target groups (gender, ethnicity, age and/or other relevant characteristics).

Please indicate the strong and weak(er) points.

The step-wise RCT will focus on abdominal surgeries. The proposal clearly articulates the objectives, inclusion criteria, outcome measures and statistical analyses to be undertaken. Sample size calculations are provided. Patient orientation is ensured through active involvement of a patient group.

There was not much discussion about the design of the guidance services provided through the portal. I think this is critical for success. Possibly, much of the structural details of the nature of services to be provided through the portal has already been developed in the previous pilot that focused on gynecological surgery. Nonethless, attention needs to be given to this since different surgeries are focused on in this sty.

I was wondering if consideration should be given to certain substrata within the population group that may be less well serviced by new approaches to providing guidance through the internet-- e.g., elderly, non-Dutch/English speakers, individuals in lower socioeconomic groups. It would be good to give attention to some of these sub-populations in your effectiveness analysis.

2.2 Cost-effectiveness analysis

+	+/-	-
X		

The purpose of the cost-effectiveness analysis (CEA) is to assess the proposed gain in health care efficiency of the (new) intervention(s) compared to the usual care provided in the Netherlands.

Consider:

- clarity;
- consistent with the objectives and research questions?
- well-designed, considering (new) intervention versus usual care in the Netherlands (reference), time horizon, effect parameters, etc.?
- are all relevant effects and costs assessments included?
- appropriate data collection?
- will results be applicable in other relevant settings?

Please indicate the strong and weak(er) points.

A well conceptualized cost-effectiveness analysis is at the centre of the study. Appropriate data collection and analytic plans have been articulated. The study will most definitely be applicable to other areas in the Netherlands, as well as internationally. There is clear potential for substantial efficiency gains in health care provision.

The last paragraph in the costs-effectiveness analysis section on page 10 captures all the the standardized approaches used in the past in this area, but it seems rote.

The analysis will be performed at the hospital and societal level. I think the patient and family level should also be taken into consideration. I would imagine that the move towards greater use of day surgery and one-day hospital stays is pushing off more of the care giving roles and costs to family and community. These costs should be captured in the analysis. Since the study is focused on efficiency, it would be innovative to try to capture a broader set of societal costs, particularly individual and family time use and out of pocket costs, rather than narrowly focusing on system and paid-labour productivity loss costs.

2.3 Budget impact analysis

+	+/-	-
X		

The purpose of the budget impact analysis (BIA) is to assess the financial consequences of dissemination of the (new) intervention(s). Information obtained from the BIA can be used for policy decisions on a national, regional and/or local level. Various perspectives can be taken into account; the government and insurance perspectives must always be considered.

Consider:

- clarity;
- well-designed in terms of perspectives, scenarios, population, time horizon, etc.?
- are all relevant cost assessments included?
- appropriate data collection?

Please indicate the strong and weak(er) points.

A clear budget impact analysis has been provided with plans for sensitivity analysis under different use/service provision scenarios. Consideration is given to a broad roll out of the intervention across the Netherlands. The government and insurer perspective are considered, but not the patient and community perspective (see my comment on the cost-effectiveness analysis section).

2.4 Systematic review

+	+/-	-
	X	

See 'Strategy' and 'Tables systematic review' (if available)

Consider:

- selection of search terms;
- are all relevant databases included?
- selection of papers;
- are any references relevant to this specific proposal missing?
- are the conclusions of the systematic review justified?

Please indicate the strong and weak(er) points.

A lot of attention (text space in the proposal) was given to a systematic review that was undertaken in April 2013 to identify evidence on the effectiveness of ICT and/or ehealth applications. Though the review search strategy and terms seem acceptable-- focusing only on patient and intervention variables of the PICO search framework--the focus on RCTs seems narrow. I imagine this is a thin literature and there is much to be learned from non-RCT studies. Also, though the authors state that they are not able to conduct a best evidence synthesis, they do end up making a best evidence synthesis type statement.. "we concluded that ehealth or other forms of ICT and telehealth probably improves clinical patient outcomes, patient knowledge and patient satisfaction..."

The authors also note that no cost effectiveness study was found, yet the objective did not included consideration of cost-effectiveness, only effectiveness.

2.5 Feasibility

+	+/-	-
X		

See 'Strategy' and 'Inclusion feasibility'

Consider:
- realistic phasing and timetable.
- prospects of achieving the objective(s) using this strategy;
- research protocol;
- realistic number of patients/institutes/organisations;
- recruitment of patients.

Please indicate the strong and weak(er) points.

The timetable and phasing are feasible, and it is clear that the team has the ability to execute the study as described.

Overall the feasibility is very high. But I would encourage the authors to consider "pushing the margins" a little on the costs to be considered-- specifically including individual and community time costs and out of pocket costs. I would also encourage them to focus on some marginal populations that may be less well served by the ehealth technology.

Lastly, though there was some discussion about the long-term management of the portal, it was not clearly stated who currently owns and manages it, and how potential financial gains will be dealt with if the portal does indeed become widespread across the Netherlands. Will insurers save money or potentially make higher profits as a result of the innovation? These issues need to be sorted out in advance, especially since there is substantial public R&D funds being invested in its development.

3. Project group

Legenda: + (+), +/- (+/-), - (-)

3.1 Project group

+	+/-	-
X		

Consider:
- relevant expertise and disciplines;
- familiarity with research area;
- prior activities and products.

Please indicate the strong and weak(er) points.

It is a strong project group. There are a number of participants with clinical backgrounds, which is important for this kind of study. What about a sociologist? Such expertise might provide a broader understanding of social

implications of new technologies. Some in depth interviews could be undertaken by such a person to see how the technology affects social connectedness and integration.

The team has a good tack record in this area of research, particularly the principal investigator.

4. Overall quality assessment

Legenda: VG (Very good), G (Good), S (Sufficient), F (Fair), P (Poor)

4.1 Overall quality assessment

VG	G	S	F	P
X				

Give the overall quality assessment about the grant application (regarding points 1-3). Please indicate the most important strong and weak(er) points.

It is a solid proposal with a team that has a good track record in this area of inquiry.

I would encourage the team to "push the margins" in terms of the economic evaluation component, rather than falling back on the standard approach they and others have taken in previous studies. After all, this is a academic endeavor, so innovation should be undertaken not only through the technology being evaluated, but the evaluation methods themselves. Also, I would encourage the team to give focus to vulnerable populations in their study.

The systematic review needs more work. Possibly consider a broader set of inclusion criteria, and formalize the synthesis approach.

As noted, the proposal has many grammatical errors and the use of acronyms becomes a challenge for readers particularly since some key acronyms are not defined.

5. Additional value to current knowledge

Legenda: + (+), +/- (+/-), - (-)

5.1 Additional value to current knowledge

+	+/-	-
X		

Consider:

- will this project yield new information?;
- ensure it does not duplicate past or ongoing projects.

Please indicate the strong and weak(er) points.

This project will yield new information. At one level, it is building on a previous study on gynecological surgery, but it is new and different enough to warrant academic funding. The next iteration of the web portal may be able to be developed by the heath care system once this study irons out all of the details through a trial focused on abdominal surgery.

6. Budget

Legenda: TH (Too high), R (Realistic), TL (To low), NJ (No judgement)

6.1 Budget

TH	R	TL	NJ
			X

Please give your judgement of the budget based on the data supplied in the grant application. Important for the judgement of the budget is that usual care covered by Dutch health insurance is not chargeable to the grant. There is a comprehensive overview of the requested budget (in Dutch).

If you are not able to assess the requested budget please type 'No Judgement' in the textbox.

I am unable to assess, as many of the item descriptions are in Dutch.
