# Supplementary material for: Substitution of Usual Perioperative Care by eHealth to Enhance Postoperative Recovery in Patients Undergoing General Surgical or Gynecological Procedures: Study Protocol of a Randomized Controlled Trial
Source: JMIR Res Protoc. 2016 Dec 21;5(4):e245. doi: 10.2196/resprot.6580 (PMC5215129; doi:10.2196/resprot.6580)
Supplement: Multimedia Appendix 4 [file resprot_v5i4e245_app4.pdf]

Subsidieprogramma / Subsidy programme	:	**DoelmatigheidsOnderzoek 2013-2015**
Dossiernummer / Dossier number	:	**80-83700-98-42074**
Aanvrager / applicant	:	**Prof. dr. J.R. Anema MD PhD**
Projecttitel / Project title	:	**SUBSTITUTION OF USUAL PERIOPERATIVE CARE BY E-HEALTH & ICT: A cost-effectiveness analysis alongside a stepped wedge cluster randomised controlled trial**
Beoordelingscode / Assessment code	:	**B.2013.01403**

1. Objective and problem definition

Legenda: + (+), +/- (+/-), - (-)

1.1 Objective and problem definition

+	+/-	-
X		

Consider:

- how clear and specific is the objective?;
- how clear and verifiable are the problem definition and hypothesis and is it consistent with the objective?;

Please indicate the strong and weak(er) points.

The objective of the application is plain and clear: an eHealth application with proven effectiveness will be tested on cost-effectiveness. The problem definition is straightforward and convincing. Current practice (usual perioperative care), however, could be described better. Now only the disadvantages of usual care are highlighted. The way it works remains unexplained. The impression is given that usual perioperative care is more or less empty, so it is not evident which elements of usual care are "substituted". The intervention seems to work out as additional care rather than substitution of care.
The intervention itself is described only briefly. Throughout the application several details of the intervention emerge, but it remains unclear how tight the application has been (or will be) integrated with current practice and current information systems.

2. Strategy

Legenda: + (+), +/- (+/-), - (-)

2.1 Clinical study

+	+/-	-
X		

Consider:

- clarity;
- adequacy in terms of problem definition;
- adequacy of study design, outcome parameters (patient oriented), sample size calculation and analyses;
- relevant differences within target groups (gender, ethnicity, age and/or other relevant characteristics).

Please indicate the strong and weak(er) points.

The overall study design is very strong. A stepped wedge design is a proper choice for such incremental implementation programme.

Yet, some details need to be explained:
- Are patients without internet access excluded from the study? I guess so, but it has not been explicited.
- What do the applicants mean with "partial" substition?
- Will Webportal be the only medium for the communication of monitoring and transition information? Does it mean that conventional media like discharge letters are abandonned?
- How is current peri-operative care organised? Is there significant variation between the centres in the study? If so, how do the researchers correct for these differences?
- Whose protocol adherence will be measured? The patients'? The health-care providers'? Or both?

2.2 Cost-effectiveness analysis

+	+/-	-
X		

The purpose of the cost-effectiveness analysis (CEA) is to assess the proposed gain in health care efficiency of the (new) intervention(s) compared to the usual care provided in the Netherlands.

Consider:

- clarity;
- consistent with the objectives and research questions?
- well-designed, considering (new) intervention versus usual care in the Netherlands (reference), time horizon, effect parameters, etc.?
- are all relevant effects and costs assessments included?
- appropriate data collection?
- will results be applicable in other relevant settings?

Please indicate the strong and weak(er) points.

The CEA plan looks rigorous. The choice to imputate missing data is defendable in the context of repeated measurements data.

2.3 Budget impact analysis

+	+/-	-
X		

The purpose of the budget impact analysis (BIA) is to assess the financial consequences of dissemination of the (new) intervention(s). Information obtained from the BIA can be used for policy decisions on a national, regional and/or local level. Various perspectives can be taken into account; the government and insurance perspectives must always be considered.

Consider:

- clarity;
- well-designed in terms of perspectives, scenarios, population, time horizon, etc.?
- are all relevant cost assessments included?
- appropriate data collection?

Please indicate the strong and weak(er) points.

The BIA plan also looks rigorous. A rich variety of scenario's will be evaluated against a 5-years time perspective.

2.4 Systematic review

+	+/-	-
X		

See 'Strategy' and 'Tables systematic review' (if available)

Consider:

- selection of search terms;
- are all relevant databases included?
- selection of papers;
- are any references relevant to this specific proposal missing?
- are the conclusions of the systematic review justified?

Please indicate the strong and weak(er) points.

Adequate setup for a literature study. Although it is strange that 1456 hits from the systematic search resulted in only 13 eligible studies. As a consequence, the definite selection of studies leans heavily on the exclusion process. Which could have been beter specified than only mentioning that "mainly" two criteria were used (not peri-operative, not RCT).

2.5 Feasibility

+	+/-	-
X		

See 'Strategy' and 'Inclusion feasibility'

Consider:

- realistic phasing and timetable.
- prospects of achieving the objective(s) using this strategy;
- research protocol;
- realistic number of patients/institutes/organisations;
- recruitment of patients.

Please indicate the strong and weak(er) points.

Time schedule is feasible. First year may be generous, although the time needed for optimisation webportal is difficult to estimate. It is not specified how rigorous this optimisation will be. Is it only new content knowledge that must be added? Or should the application code be revised?
Second and third year have a time schedule enforced by protocol.
Last year seems generous, with only writing work.

Research objectives are realistic and achievable.

Research protocol seems feasible, athough there is no estimation of time needed for the patient to fill out all questionnaires included in the plan.

Recruitment of organisations is feasible, as already enough letters of intention have been signed.

Patient recruitment is very feasible, as prior experience has shown.

3. Project group

Legenda: + (+), +/- (+/-), - (-)

3.1 Project group

+	+/-	-
X		

Consider:

- relevant expertise and disciplines;
- familiarity with research area;
- prior activities and products.

Please indicate the strong and weak(er) points.

All relevant expertise is present at the highest level of qualification.

4. Overall quality assessment

Legenda: VG (Very good), G (Good), S (Sufficient), F (Fair), P (Poor)

4.1 Overall quality assessment

VG	G	S	F	P
	X			

Give the overall quality assessment about the grant application (regarding points 1-3). Please indicate the most important strong and weak(er) points.

Strong points:
1. The proposed intervention is a promising contribution to a relevant problem that affects the quality of care.
2. The intervention has enough been piloted and studied to give confidence that a broader implementation and a cost-effectiveness study will be successful.
3. The project group is well equipped to manage this project.

Weak points:
1. The application text is not clear about the baseline condition. The contrast between control and intervention condition should be further specified.
2. The application text is not clear about the purpose of adaption of Webportal. As a consequence, it is difficult to make a judgment about the effort (time, cost) needed for "optimisation".

5. Additional value to current knowledge

Legenda: + (+), +/- (+/-), - (-)

5.1 Additional value to current knowledge

+	+/-	-
X		

Consider:

- will this project yield new information?;
- ensure it does not duplicate past or ongoing projects.

Please indicate the strong and weak(er) points.

Yes, this project has indeed additional value to current knowledge. I am not aware of duplicate projects. Strong point is the fact that the proposed study is both an evaluation of cost-effectiveness and a first step towards nation-wide implementation of the intervention. to this end, the collaboration with patient platform, scientific board and insurance company is a promising condition.

6. Budget

Legenda: TH (Too high), R (Realistic), TL (To low), NJ (No judgement)

6.1 Budget

TH	R	TL	NJ
			X

Please give your judgement of the budget based on the data supplied in the grant application. Important for the judgement of the budget is that usual care covered by Dutch health insurance is not chargeable to the grant. There is a comprehensive overview of the requested budget (in Dutch).

If you are not able to assess the requested budget please type 'No Judgement' in the textbox.

The development costs for adaptation of Webportal cannot be judged, because there is no information about what should be achieved.
The reservation of one whole year only for writing seems generous.
