# Supplementary material for: Substitution of Usual Perioperative Care by eHealth to Enhance Postoperative Recovery in Patients Undergoing General Surgical or Gynecological Procedures: Study Protocol of a Randomized Controlled Trial
Source: JMIR Res Protoc. 2016 Dec 21;5(4):e245. doi: 10.2196/resprot.6580 (PMC5215129; doi:10.2196/resprot.6580)
Supplement: Multimedia Appendix 5 [file resprot_v5i4e245_app5.pdf]

Subsidieprogramma / Subsidy programme	:	**DoelmatigheidsOnderzoek 2013-2015**
Dossiernummer / Dossier number	:	**80-83700-98-42074**
Aanvrager / applicant	:	**Prof. dr. J.R. Anema MD PhD**
Projecttitel / Project title	:	**SUBSTITUTION OF USUAL PERIOPERATIVE CARE BY E-HEALTH & ICT: A cost-effectiveness analysis alongside a stepped wedge cluster randomised controlled trial**
Beoordelingscode / Assessment code	:	**B.2013.01404**

1. Objective and problem definition

Legenda: + (+), +/- (+/-), - (-)

1.1 Objective and problem definition

+	+/-	-
	X	

Consider:

- how clear and specific is the objective?;
- how clear and verifiable are the problem definition and hypothesis and is it consistent with the objective?;

Please indicate the strong and weak(er) points.

The objective is clear and quite specific.

It should be better outlined, however, that this study will not replace traditional perioperative care completely with modern communication techniques, but will only partially replace it.

Furthermore the term "EHealth & ICT" used throughout is confusing and not specific. More functional terms like "electronic consultation" are much better suited for the purpose here. When a short term would be needed my suggestion would be to use eHealth, with a sharp definition of eHealth in the introduction. ICT cannot be a goal, and in modern times (nearly) all information is handled by ICT. So, the term ICT is non-desciptive, whereas eHealth, or rather "electronic consultation" and related terms have relevance to patients and health professionals.

2. Strategy

Legenda: + (+), +/- (+/-), - (-)

2.1 Clinical study

+	+/-	-
X		

Consider:

- clarity;
- adequacy in terms of problem definition;
- adequacy of study design, outcome parameters (patient oriented), sample size calculation and analyses;
- relevant differences within target groups (gender, ethnicity, age and/or other relevant characteristics).

Please indicate the strong and weak(er) points.

The study is well designed.

2.2 Cost-effectiveness analysis

+	+/-	-
X		

The purpose of the cost-effectiveness analysis (CEA) is to assess the proposed gain in health care efficiency of the (new) intervention(s) compared to the usual care provided in the Netherlands.

Consider:

- clarity;
- consistent with the objectives and research questions?
- well-designed, considering (new) intervention versus usual care in the Netherlands (reference), time horizon, effect parameters, etc.?
- are all relevant effects and costs assessments included?
- appropriate data collection?

- will results be applicable in other relevant settings?

Please indicate the strong and weak(er) points.

OK

2.3 Budget impact analysis

+	+/-	-
X		

The purpose of the budget impact analysis (BIA) is to assess the financial consequences of dissemination of the (new) intervention(s). Information obtained from the BIA can be used for policy decisions on a national, regional and/or local level. Various perspectives can be taken into account; the government and insurance perspectives must always be considered.

Consider:

- clarity;
- well-designed in terms of perspectives, scenarios, population, time horizon, etc.?
- are all relevant cost assessments included?
- appropriate data collection?

Please indicate the strong and weak(er) points.

OK

2.4 Systematic review

+	+/-	-
X		

See 'Strategy' and 'Tables systematic review' (if available)

Consider:

- selection of search terms;
- are all relevant databases included?
- selection of papers;
- are any references relevant to this specific proposal missing?
- are the conclusions of the systematic review justified?

Please indicate the strong and weak(er) points.

OK

2.5 Feasibility

+	+/-	-
	X	

See 'Strategy' and 'Inclusion feasibility'

Consider:

- realistic phasing and timetable.
- prospects of achieving the objective(s) using this strategy;
- research protocol;
- realistic number of patients/institutes/organisations;
- recruitment of patients.

Please indicate the strong and weak(er) points.

The procedure for selecting patients is OK, the numbers seem realistic and achievable.
What is missing is attention for the inclusion of the study into the daily clinical activities within the hospitals. Especially attention is missing for the role clinical medical specialists have to take in the study, and the way they are expected to access the information and communicate with the patients.
It is a well known fact that specialists who do their daily work in an Electronic Health Record (EHR) environment are not easily motivated to log on to a different (web) application for special reasons like this study, to enter patient identification again, and to not have a connection between the EHR context and the eConsult context. Especially this remark is relevant since it is my assumption that "tradionional" post-operative care is not in all cases and al situations replaced completely by eHealth solutions.

Also, on a more technical level, attention is missing for possible problems in the connection/integration of the activities in the electronic environment of the hospitals. There is always a possibility that a hospital's IT staff sees problems on a technical, semantics or security level, and then the expertsie within the group to deal with these problems is missing, it seems. See the "Project Group" section.

3. Project group

Legenda: + (+), +/- (+/-), - (-)

3.1 Project group

+	+/-	-
	X	

Consider:

- relevant expertise and disciplines;
- familiarity with research area;
- prior activities and products.

Please indicate the strong and weak(er) points.

The expertise in the clinical and social medicine areas is adequate.
It is a bit amazing that no person(s) with a medical informatics background are included in the team. Two roles can be seen: the integration of the activities for this project into the daily digital clinical life of the medical specialists (and others), and the preparation for future integration of the ways of working developed here into routine clinical practice.

4. Overall quality assessment

Legenda: VG (Very good), G (Good), S (Sufficient), F (Fair), P (Poor)

4.1 Overall quality assessment

VG	G	S	F	P
		X		

Give the overall quality assessment about the grant application (regarding points 1-3). Please indicate the most important strong and weak(er) points.

This is a grant application on a highly relevant subject, yielding possibly better and more efficient solutions for peri-operative care. The group has a lot of experience in the evaluation of such interventions in established routine clinical practices.

The point of concern is the relative shortage of attention for the nature of the alternative solution for perioperative care, presented here, based upon eHealth. The group is missing expertise in medical informatics, and thus a risk exists that certain problems during the study possibly cannot be overcome, but, moreover, that sustainability of the results might be insufficiently guaranteed.

It is well known that one of the most problematic points in eHealth development is the future integration of successful study results into routine clinical practice. That is what is usually meant by sustainability in this area. The authors apparently are aware of this, but this awareness is limited to the sentences on page 13, I cite:

"
Our intention is integration or linking the portal to existing hospital systems and national registration systems to facilitate the implementation. The provider of the web portal works therefore with open standards to make the portal compatible with systems within hospitals.
"

The bare statement that the provider uses standards is by no means a guarantee for future interoperability. Moreover, this strategic issue should not be left to the responsability of the eHealth provider, but taken in the hands by the consortium itself.

A last over-all comment on quality is the use of the English language in this grant application. The quality of the English is so poor, and errors are so frequent, that on certain places ambiguity occurs.
The authors should be strongly advised to avoid this in future publications.

5. Additional value to current knowledge

Legenda: + (+), +/- (+/-), - (-)

5.1 Additional value to current knowledge

+	+/-	-
X		

Consider:

- will this project yield new information?;
- ensure it does not duplicate past or ongoing projects.

Please indicate the strong and weak(er) points.

This study will yield new knowledge on substitution of regular peri-operative care by eHealth solutions.

6. Budget

Legenda: TH (Too high), R (Realistic), TL (To low), NJ (No judgement)

6.1 Budget

TH	R	TL	NJ
	X		

Please give your judgement of the budget based on the data supplied in the grant application. Important for the judgement of the budget is that usual care covered by Dutch health insurance is not chargeable to the grant. There is a comprehensive overview of the requested budget (in Dutch).

If you are not able to assess the requested budget please type 'No Judgement' in the textbox.

The budget seems realistic
